# Supramolecular Organic Framework with Multidimensional Storage Spaces for Ultrahigh-Capacity Iodine Capture from Seawater

**DOI:** 10.34133/research.0608

**Published:** 2025-02-07

**Authors:** Lijuan Feng, Jun Zhang, Jiacheng Zhang, Xuewen Cao, Zhanhu Guo, Yihui Yuan, Ning Wang

**Affiliations:** ^1^ State Key Laboratory of Marine Resource Utilization in South China Sea, Collaborative Innovation Center of Marine Science and Technology, Hainan University, Haikou 570228, P. R. China.; ^2^ Department of Mechanical and Civil Engineering, Faculty of Engineering and Environment, Northumbria University, Newcastle Upon Tyne NE1 8ST, UK.

## Abstract

Given the important role of iodine resources in chemical industry application and the scarcity of geogenic iodine resources, sustainable access to iodine resources has become increasingly crucial. Seawater is the largest iodine reservoir on Earth, but efficient chemical methods for recovering iodine from seawater are still lacking. Concurrently, the remediation of radioactive iodine pollution in seawater, caused by nuclear accident, remains a great challenge. Supramolecular organic frameworks (SOFs) are considered promising candidates for the recovery of aqueous iodine. However, currently available SOF adsorbents lack sufficient iodine storage space, resulting in low iodine adsorption capacity. Herein, we developed a 3-dimensional (3D) SOF, named SOF-HTNI, via the self-assembly of 2 adjustable compounds, including the internal amine bond-rotatable 5-(bis(4-carboxybenzyl)amino)isophthalic acid (HT) and the configuration-transformable 4,4′-[1,4-phenylenedi-(1E)-2,1-ethenediyl]bis-pyridine (NI), for highly efficient iodine recovery from seawater. Due to the rigid support and the formation of hydrogen bonds and π–π stacking interactions between the compounds, interconnected 1D channels and 2D interlayer nanospaces are constructed within SOF-HTNI, providing abundant flexible spaces for iodine storage. By combining the charge interaction of the amine and pyridyl groups from the compounds with the binding ability of aromatic rings, SOF-HTNI achieves impressive iodine adsorption capacities of 436.56 mg g^−1^ to iodide and 5.03 g g^−1^ to triiodide. Notably, SOF-HTNI realizes a high iodine capture capacity of 46 mg g^−1^ in natural seawater, 40 times greater than that of seaweed. These findings make SOF-HTNI a highly promising material for iodine pollution treatment and iodine resource recovery in seawater.

## Introduction

Iodine and its derivatives play essential roles in numerous applications, including catalysis, medicine, and industry [1,2]. However, iodine ranks only 61st among all the elements in terms of abundance, making it one of the least abundant nonmetallic elements on Earth. Recently, the global demand for iodine has been consistently surging, leading to a corresponding increase in its acquisition cost [3]. Despite this, seawater holds the world’s largest reserve of iodine, accounting for approximately 70% of the total iodine quantity, with iodide and iodate being the dominant iodine species in seawater, at approximate concentration of 28 parts per billion (ppb) and 36 ppb, respectively [4,5]. Seaweed, which grows in seawater, accumulates a substantial amount of iodine [6]. The extraction of iodine from seaweed precedes the development of iodine extraction from caliche ore and underground brine, and it continues to serve as an important method for acquiring iodine resources [7]. However, extracting iodine from seaweed faces several challenges, including the ultralow concentration of iodine in seawater and the adverse environmental effects of large-scale seaweed aquaculture. The extraction of iodine from seawater using chemical adsorbents is considered a promising alternative to iodine extraction from seaweed cultivation; however, such techniques are still lacking. Additionally, iodine is a high fission yield product of nuclear energy industry, and nuclear accidents have caused the leakage of large quantities of radioactive iodine, including ^129^I and ^131^I, into seawater, mainly in form of I_2_ [7,8]. With a prolonged half-life (1.6 × 10^7^ years for ^129^I), high solubility, rapid diffusion, and the ability to participate in biological processes, radioactive iodine poses a long-term threat to the marine environment and human health [9,10]. However, strategies for removal of radioactive iodine from the seawater environment are still lacking. Hence, there is an urgent need to design and develop efficient chemical techniques for accessing seawater iodine resources and for managing radioactive iodine pollution in seawater.

Crystalline porous frameworks have garnered considerable attention owing to their potential applications in adsorption, storage, and separation [11–16]. Supramolecular organic frameworks (SOFs) are crystalline porous frameworks constructed through weak noncovalent interactions, such as hydrogen bonding and π–π stacking [17,18]. Compared to the other crystalline porous frameworks, SOFs possess abundant active sites, flexible storage space, and enhanced reversibility, making them more suitable to storing target substances [19,20]. Furthermore, SOFs are characterized by better polarity and hydrophilicity, which make them favorable for use in water environments. However, during the construction of storage spaces, the weak strength and flexibility of hydrogen bonds, along with the planar stacking of π–π interactions, often lead to the formation of 2-dimensional (2D) layered structures [21,22]. Notably, 3D structures can provide interconnected spaces that are favorable for the transport of substances, benefiting their adsorption performance [23,24]. The first 3D SOF was assembled by encapsulating macrocycles at the head-to-tail positions of the organic compound [25]. Subsequent efforts have led to the construction of 3D SOFs using strategies such as introducing multiple noncovalent bonding interactions and enhancing structural cross-linking [26,27]. Therein, the strategy of noncovalent bonding interactions by generating π stacking interactions can not only significantly improve the stability of adsorbents in water but also facilitate the enhancement of iodine adsorption performance [9]. The challenge of this method lies in the need for both strong interactions between the compounds to stabilize the structure and the mutual adjustability of the compounds to facilitate the self-assemble process [28]. Therefore, flexible and self-adjustable organic compounds with semirigid structures are desirable for synthesis of advanced 3D SOF materials with abundant storage space for aqueous iodine capture [29].

**Fig. 1.**
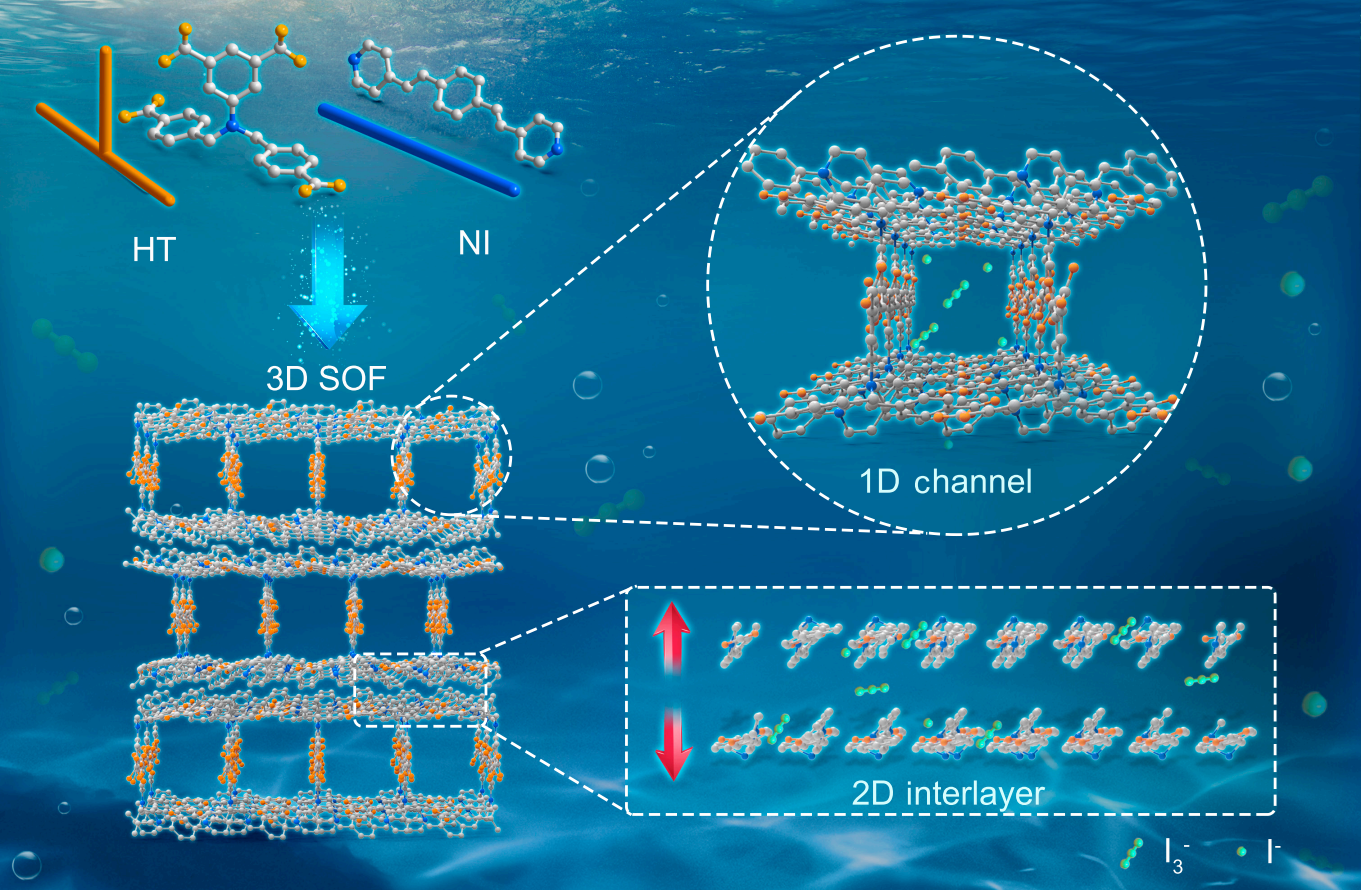
Schematic diagram illustrating the self-assembly of SOF-HTNI and its ability to capture triiodide and iodide from seawater.

Herein, we present the fabrication of a novel 3D SOF, designated as SOF-HTNI, achieved through the interaction of 2 spatially adjustable compounds, the T-pattern 5-(bis(4-carboxybenzyl)amino)isophthalic acid (HT) and the I-pattern 4,4′-[1,4-phenylenedi-(1E)-2,1-ethenediyl]bis-pyridine (NI). Attributing to the charge interactions of amine and pyridyl groups, as well as the binding ability of aromatic rings, SOF-HTNI exhibits selective adsorption ability for triiodide in simulated nuclear polluted seawater and for iodide in natural seawater, making it suitable for recovering iodine from seawater. Furthermore, the 3D structure of SOF-HTNI contains interconnected 1D channels and 2D interlayer spaces to provide ample iodine storage space, enabling SOF-HTNI to realize a high iodine adsorption capacity. The findings of this study establish an effective strategy for fabricating multidimensional 3D SOF materials and also present a promising material for iodine pollution treatment and iodine resource recovery from seawater (Fig. 1).

## Results

### Design and synthesis principle

The design of target structure using appropriate compounds is crucial for the self-assembly of functional frameworks [30]. The HT is an amine-linked carboxylic compound, featuring flexible oriented carboxylic groups and a semi-rigid support structure. The NI contains abundant pyridine groups and has a convertible configuration that can change from E to Z under heating. HT and NI have complementary carboxylic and pyridine groups, as well as similar molecular lengths, which favor the self-assembly of a regular framework. Additionally, both the amine group of HT and the pyridine group of NI can bind iodine with high affinity. Therefore, HT and NI compounds were employed to construct a 3D SOF material using solvothermal method [31].

### Characterization of 3D SOF-HTNI

**Fig. 2.**
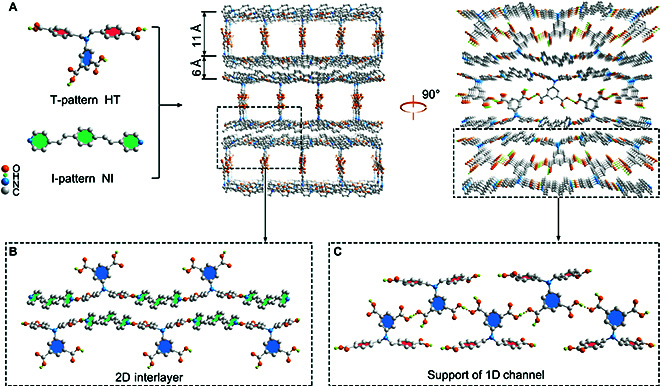
Schematic illustration for the architecture of 3D SOF-HTNI. (A) Self-assembly between the compounds HT and NI to form the 3D structure of SOF-HTNI. (B) The 2D interlayer structure in SOF-HTNI, constructed by NI and the main chain of HT. (C) Support of the 1D channel in SOF-HTNI, facilitated by the interactions of branch chains from HT.

The characteristics of SOF-HTNI were investigated to reveal its chemical properties. Single-crystal x-ray diffraction (SC-XRD) analysis indicated that the crystal structure of SOF-HTNI belonged to the P2_1_/c space group, with each asymmetric unit containing one HT and one NI (Fig. 2A and Tables S1 to S4). During synthesis, the HT underwent C–N bond rotation, while the NI transformed from its E configuration to Z configuration, contributing to the assembly of the 3D SOF material. The main chain of HT, with a matching functional group and similar chain length to the linear NI, facilitated the formation of a 2D interlayer structure through intermolecular hydrogen bonding and π–π stacking interactions (Fig. 2B). Additionally, the branch chains of HT, with participated in intermolecular hydrogen bonding interactions between carboxyl groups on rigid benzene rings, formed 1D channels that supported the creation of the 3D structure (Fig. 2C). Consequently, the supramolecular architecture, stabilized by hydrogen bonding and π–π stacking interactions, featured interconnected 1D channels and 2D interlayer spaces within the 3D network. The 1D channel measured 11 Å × 9 Å, and the 2D interlayer space had a thickness of 6 Å, offering substantial potential flexible spaces for iodine storage (Fig. S1) [32]. The abundant flexible spaces, on one hand, facilitate the exposure of functional sites for iodine binding and, on the other hand, promote the transport of iodine within the material, thereby contributing to its iodine adsorption performance.

XRD analysis of SOF-HTNI confirmed the pure phase of the synthesized SOF-HTNI, which aligned well with simulated data from SC-XRD analysis (Fig. S2). Thermogravimetric and derivative thermogravimetric (TG-DTG) analyses demonstrated a rapid weight loss of 44% at temperatures between 300 and 400 °C, corresponding to the decomposition of the compounds, thereby confirming the good thermal stability of SOF-HTNI (Fig. S3). Scanning electron microscopy (SEM) revealed clustered needle-shaped crystals of SOF-HTNI (Fig. S4). Energy-dispersive spectroscopy (EDS) mapping analysis confirmed the homogeneous distribution of elements C, N, and O in SOF-HTNI. Nitrogen adsorption–desorption analysis indicated a gas absorption amount of 20.59 cm^3^ g^−1^ at standard temperature and pressure (STP), with a Brunauer–Emmett–Teller (BET) specific surface area of 18.59 m^2^ g^−1^ at 77 K (Fig. S5) [25]. The pH stability assessment was conducted by soaking SOF-HTNI in aqueous solutions with pH values ranging from 3 to 10 for 2 d. XRD analysis showed that the adsorbent retained its crystal structure, confirming the high stability of SOF-HTNI under diverse pH conditions (Fig. S6). The hydrophilicity of the material is crucial for iodine adsorption in an aqueous environment. The hydrophilicity was determined through a water contact angle test, which indicated a moderate hydrophilicity with a contact angle of 63.51° (Fig. S7). This moderate hydrophilicity results from the balance between hydrophilic and hydrophobic components within the structure, which allows the material to effectively capture both I^−^ and I_3_^−^ in aqueous phase.

### Iodine uptake ability of SOF-HTNI

**Fig. 3.**
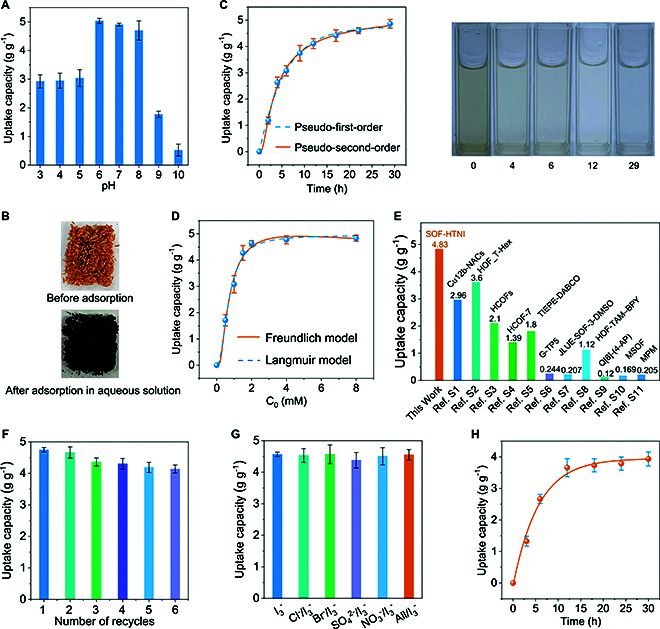
Adsorption performance of SOF-HTNI for triiodide. (A) Influence of pH on I_3_^−^ adsorption capacity. (B) Morphology of SOF-HTNI before and after I_3_^−^ loading. (C) Adsorption kinetic of SOF-HTNI for I_3_^−^ in aqueous solution, accompanied by the photographs showing the color changes of the solution. (D) Adsorption isotherm of SOF-HTNI for I_3_^−^ in aqueous solution. (E) Comparison of iodine uptake capacity of SOF-HTNI for I_3_^−^ with various noncovalent organic frameworks. (F) Reusability of SOF-HTNI. (G) Anti-interfering ability of SOF-HTNI in adsorbing I_3_^−^ in the presence of competing anions. (H) Uptake capacity of SOF-HTNI for iodine in I_3_^−^ ion-spiked natural seawater.

The iodine uptake ability of SOF-HTNI was evaluated for both vapor-phase I_2_ as well as aqueous I_3_^−^, I^−^, and IO_3_^−^. In the vapor phase, SOF-HTNI was exposed to iodine vapor at 75 °C for 24 h, resulting in a color change from yellow to black and an equilibrium iodine uptake capacity of 715 mg g^−1^ (Fig. S8). In aqueous conditions, the uptake abilities of SOF-HTNI toward I_3_^−^ were evaluated in I_2_/KI solution, and the highest adsorption capacity of 5.03 g g^−1^ was achieved at pH 6.0 (Fig. 3A). After the loading of I_3_^−^, the color of SOF-HTNI transformed from yellow to black, confirming that SOF-HTNI exhibits good adsorption performance for I_3_^−^ in aqueous conditions (Fig. 3B and Fig. S9). SOF-HTNI exhibits better adsorption performance in aqueous conditions than in the vapor phase, which can be attributed to the presence of unique functional sites and their selective interactions with the dominant iodine species in aqueous environments [33,34]. The analysis of pH-dependent experiments of SOF-HTNI for I_3_^−^ also showed decreased capacities at pH values higher than 8 and lower than 6, which can be attributed to the effect of pH on the adsorbent interface and iodine species in the aqueous solution. The analysis of the adsorption kinetics of SOF-HTNI for I_3_^−^ revealed that an equilibrium adsorption capacities of 4.86 g g^−1^ was achieved within 29 h (Fig. 3C). The analysis of the adsorption isotherm showed that the adsorption behavior fitted better with the Langmuir sorption model (Fig. 3D). The maximum experimental adsorption capacity for aqueous I_3_^−^ were 4.83 g g^−1^, closely matching the calculated theoretical maximum capacity of 4.94 g g^−1^. SOF-HTNI exhibited exceptional adsorption properties for aqueous I_3_^−^ compared to the other reported noncovalent organic framework (Fig. 3E and Table S5). After the adsorption process, the I_3_^−^ ion-loaded SOF-HTNI was left in 50 ml of pure water for 10 d, and it exhibited negligible I_3_^−^ ions leakage, which indicates its excellent retention capacity for I_3_^−^ ions in aqueous solution (Fig. S10). The reusability of SOF-HTNI for adsorbing I_3_^−^ was analyzed by eluting the bound I_3_^−^ with methanol, and 88% of the initial I_3_^−^ adsorption capacity was retained after being reused for 6 times (Fig. 3F). No obvious change in the PXRD pattern was observed after recycles, suggesting the good reusability of SOF-HTNI (Fig. S11). Competition analysis between I_3_^−^ and the coexisting interfering anions, including Cl^−^, Br^−^, SO_4_^2−^, and NO_3_^−^, was conducted. The result demonstrated that SOF-HTNI maintained good selectivity for I_3_^−^ (Fig. 3G). Additionally, to evaluate the applicability of SOF-HTNI in treating I_3_^−^ pollution, natural seawater spiked with I_3_^−^ ions was used to simulate the polluted environment. The results showed that the material exhibited an uptake capacity of 3.94 g g^−1^ and a removal rate of 79% for I_3_^−^ (Fig. 3H). I_2_ is the major form of iodine released by nuclear accidents, which may form I_3_^−^ by interacting with I^−^ in seawater. These results highlight the high potential of SOF-HTNI for treating seawater iodine pollution.

**Fig. 4.**
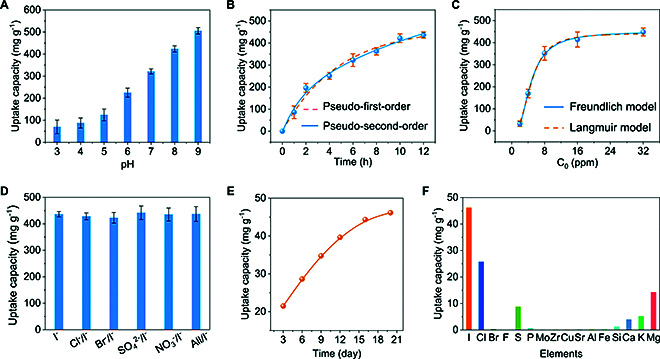
Adsorption performance of SOF-HTNI for iodide. (A) Influence of pH on I^−^ adsorption capacity. (B and C) Adsorption kinetics and adsorption isotherms of SOF-HTNI to I^−^ ions in I^−^ ion-spiked natural seawater. (D) Anti-interfering ability of SOF-HTNI in adsorbing I^−^ with the coexisting of competing anions. (E) Extraction performance of SOF-HTNI for iodine in natural seawater. (F) Selectivity of SOF-HTNI for adsorbing iodine in natural seawater.

The adsorption abilities of SOF-HTNI for IO_3_^−^ and I^−^ were determined in natural seawater by spiking these ions. The results showed that SOF-HTNI lacked adsorption ability for IO_3_^−^ (Fig. S12). As for the adsorption ability for I^−^, the pH value of the I^−^ solution was found to significantly affect the adsorption capacity, where a high adsorption capacity of 505.35 mg g^−1^ was achieved at pH 9 (Fig. 4A and Table S6). The experimental adsorption capacity is close to the theoretical adsorption capacity of 519.79 mg g^−1^ (based on the N sites). The analysis of the adsorption kinetic revealed that a high adsorption capacity of 436.56 mg g^−1^ was achieved by SOF-HTNI in 16 ppm I^−^ ion-spiked natural seawater within 12 h (Fig. 4B). The adsorption kinetic fitted well with the pseudo-second-order model, suggesting that I^−^ was absorbed mainly by chemisorption. The adsorption isotherm of SOF-HTNI for I^−^ ions was determined in I^−^ ion-spiked natural seawater with different initial I^−^ ion concentrations. The adsorption isotherm was found to fit well with the Freundlich model, with an estimated maximum calculated adsorption capacity of 449.91 mg g^−1^ (Fig. 4C). The analysis of the anti-interfering ability showed that the coexisting of several anions, including Cl^−^, Br^−^, SO_4_^2−^, and NO_3_^−^, caused negligible influence on the adsorption of I^−^, demonstrating the high adsorption selectivity of SOF-HTNI (Fig. 4D). The I^−^ ion is one of the dominant forms of iodine in natural seawater, and these results demonstrate the high potential of SOF-HTNI for recovering iodine resources from natural seawater.

The recovery performance of SOF-HTNI for iodine in natural seawater was also assessed. Over a 20-d extraction period, SOF-HTNI exhibited a high iodine adsorption capacity of 46 mg g^−1^ (Fig. 4E). The iodine content adsorbed by SOF-HTNI was approximately 43 times higher than that in dried natural seaweed, which has an average iodine concentration of 1.07 mg g^−1^ [6]. In natural seawater, SOF-HTNI still exhibited a high adsorption selectivity for iodine. Despite the fact that the concentration of chlorine is 3.2 × 10^5^ times higher than that of iodine, the adsorption capacity of SOF-HTNI to iodine was 1.79 times greater than that for chlorine ions (Fig. 4F). The combination of the high adsorption capacity and selectivity of SOF-HTNI for iodine in natural seawater suggests that SOF-HTNI holds promise as an adsorbent for recovering iodine resources from seawater.

### Adsorption mechanism of SOF-HTNI to iodine

**Fig. 5.**
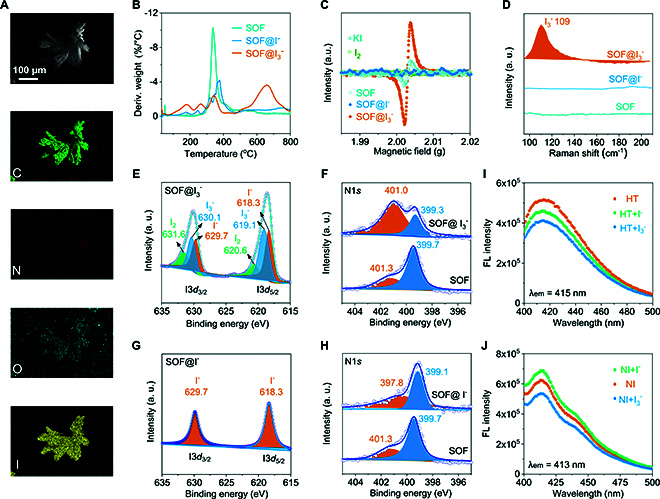
Characterization of SOF-HTNI after the adsorption of triiodide or iodide. (A) SEM image of I_3_^−^-loaded SOF-HTNI and corresponding EDS mapping analysis. (B) DTG analysis of I_3_^−^ and I^−^ ion-loaded SOF-HTNI. (C) EPR spectra of SOF-HTNI and I_3_^−^ and I^−^ ion-loaded SOF-HTNI. (D) Raman spectra of I_3_^−^ and I^−^ ion-loaded SOF-HTNI. (E and F) High-resolution XPS spectra of the iodine and nitrogen elements in I_3_^−^ ion-loaded SOF-HTNI. (G and H) High-resolution XPS spectra of the iodine and nitrogen elements in I^−^ ion-loaded SOF-HTNI. (I and J) Fluorescence spectra of NI and HT before and after their interaction with I^−^ and I_3_^−^, respectively.

The adsorption mechanism of SOF-HTNI for triiodide and iodide were analyzed in detail. SEM images revealed that after the loading of I_3_^−^, the material maintained its integrated morphology (Fig. 5A). EDS mapping showed a uniform distribution of iodine within I_3_^−^-loaded SOF-HTNI. XRD analysis indicated that diffraction peaks at 16.63°, 18.20°, and 23.34° were retained after the loading of I_3_^−^, while diffraction peaks at 12.59° and 5.53°, corresponding to distances of 7.03 and 15.98 Å within the SOF-HTNI framework, nearly disappeared (Fig. S11). The retention of these peaks suggested good π–π stacking stability of the framework, while the disappearance of the peaks may be attributed to the filling of the cavity structure in SOF-HTNI, including the 1D channel and the 2D interlayer space, by I_3_^−^ [35]. TG-DTG results showed that, compared to unused SOF-HTNI, I_3_^−^- and I^−^-loaded SOF-HTNI exhibited additional weight loss peaks of 22% and 8% from 100 to 300 °C as well as 65% and 60% above 400 °C, corresponding to iodine loss in the I_3_^−^- and I^−^-loaded SOF-HTNI (Fig. 5B and Fig. S13). Electron paramagnetic resonance (EPR) spectroscopy revealed a significantly enhanced signal after I_3_^−^ binding by SOF-HTNI and significant signal attenuation after I^−^ binding, indicating strong charge transfer interactions in iodine-loaded SOF-HTNI (Fig. 5C) [36,37]. Raman spectra showed a distinct characteristic peak at 109 cm^−1^ for I_3_^−^ ions, but this peak was absent in SOF-HTNI and I^−^-loaded SOF-HTNI, confirming the adsorption of I_3_^−^ in I_3_^−^-SOF-HTNI (Fig. 5D) [38].

X-ray photoelectron spectroscopy (XPS) analysis confirmed the iodine adsorption through the appearance of peaks for I 3*d*_5/2_ and I 3*d*_3/2_ at 631.6 and 619.3 eV in I_3_^−^-loaded SOF-HTNI, and at 626.5 and 615.7 eV in I^−^-loaded SOF-HTNI (Fig. S14). The high-resolution XPS spectra of I_3_^−^-loaded SOF-HTNI showed peaks corresponding to I_3_^−^, I^−^, and I_2_, which are different forms of iodine in the I^−^/I_2_ solution, while the I^−^-loaded SOF-HTNI displayed peaks at 629.7 and 618.3 eV, attributed to bound I^−^ ions (Fig. 5E and H). The N 1s spectra revealed a shift in peaks from 401.3 and 399.7 eV to 401.0 and 399.3 eV after I_3_^−^ adsorption, and to 397.8 and 399.1 eV after I^−^ adsorption (Fig. 5F and H), suggesting involvement of the nitrogen atoms in binding iodine species [39]. The interactions between I_3_^−^ and I^−^ with the compounds HT and NI, involved in the construction of SOF-HTNI, were analyzed by examining changes in the fluorescence properties of these 2 compounds. The results demonstrated that both I^−^ and I_3_^−^ induced changes in the fluorescence intensity, further confirming charge transfer between I^−^ or I_3_^−^ and the 2 compounds (Fig. 5I and J).

**Fig. 6.**
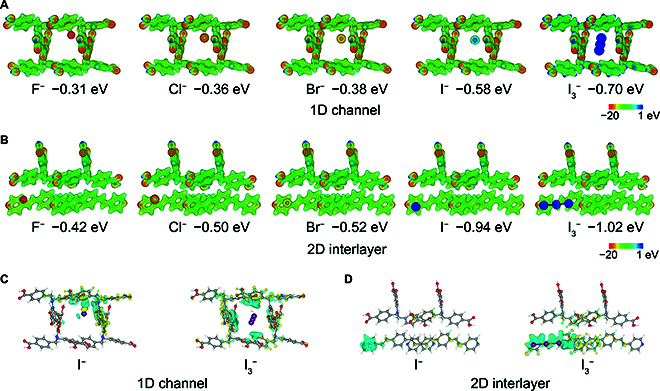
DFT calculations elucidating the binding mechanism of SOF-HTNI to I_3_^−^ and I^−^ ions. (A and B) Binding energies and ESP distributions of different ions loaded into the 1D channel and the 2D interlayer spaces, respectively. (C and D) Differential charge diagrams for the binding environment of the I^−^ and I_3_^−^ ions in the 1D channel and 2D interlayer spaces, respectively.

We further elucidated the adsorption mechanism by utilizing density functional theory (DFT) calculations. The calculated binding energies of the 1D channel within the framework for F^−^, Cl^−^, Br^−^, I^−^, and I_3_^−^ were −0.31, −0.36, −0.38, −0.58, and −0.70 eV, respectively. Correspondingly, the 2D interlayer space in the framework exhibited binding energies of −0.42, −0.50, −0.52, −0.94, and −1.02 eV for these ions, respectively (Table S7). The higher binding energies indicated a stronger adsorption ability of these structures toward I_3_^−^ and I^−^ compared to the other ions, which can be contributed to the larger ionic radii and stronger polarizability of I_3_^−^ and I^−^ ions, as well as the specific functional groups and suitable spaces within the structure, forming special anionic tunnels for the selective adsorption of I_3_^−^ and I^−^ ions [40–42]. The electrostatic potential (ESP) distribution further illustrated that the 1D channels and 2D interlayer spaces serve as theoretical functional sites for binding I_3_^−^ and I^−^ ions, owing to their electrostatic interactions (Fig. 6A and B). The calculation results also revealed that supramolecular noncovalent interactions, including hydrogen-bonding and van der Waals interactions of the aromatic rings, were responsible for the binding of I_3_^−^ and I^−^ ions (Fig. 6C and D) [43,44].

## Conclusion

In summary, this study synthesized a novel 3D SOF material, designated as SOF-HTNI, using self-adjustable compounds HT with rotatable internal amine bonds and NT with a transformable configuration, which broaden existing methods for fabricating 3D SOF materials. Through hydrogen bond interactions and π–π stacking between the compounds, interconnected 1D channels and 2D interlayer spaces were constructed within the 3D SOF, providing abundant functional spaces for storing substantial amounts of iodine. Leveraging the charge interactions of amine and pyridyl groups, along with the binding affinity of the aromatic ring, SOF-HTNI exhibited remarkable binding selectivity for I^−^ and I_3_^−^ ions over coexisting interfering anions. Owing to these advantages, SOF-HTNI demonstrated an outstanding adsorption capacity of 5.03 g g^−1^ for triiodide in an I_3_^−^ ion aqueous solution, one of the highest iodine adsorption capacities reported for noncovalent organic framework adsorbents in aqueous conditions. Furthermore, it possessed high reusability, retaining over 88% of its initial adsorption capacity after 6 reuse cycles. Additionally, SOF-HTNI achieved an impressive adsorption capacity of 436.56 mg g^−1^ for iodide in I^−^ ion-spiked seawater and achieved an ultrahigh iodine adsorption capacity of 46 mg g^−1^ in natural seawater within a 20-d extraction period. This study presents a promising adsorbent for recovering iodine resources and addressing radioactive iodine pollution in seawater.

## Materials and Methods

### Materials

Ethyl 4-(bromomethyl)benzoate (>98%), 5-aminoisophthalic acid (>98%), potassium hydroxide (>95%), hydrochloric acid (>37%), potassium iodide (>99%), and iodine (>99.8%) were obtained from Sigma-Aldrich Chemical Reagent Co. Ltd. (Shanghai, China). 4,4′-[1,4-Phenylenedi-(1E)-2,1-ethenediyl]bis-pyridine (NI) was obtained from Jilin Chinese Academy of Sciences-Yanshen Technology Co. Ltd. (Jilin, China). Deionized water was prepared in the laboratory. All chemicals were used without further purification.

### Syntheses

For synthesis of HT, the ethyl 4-(bromomethyl)benzoate (21.351 g, 0.088 mol) and potassium hydroxide (13.141 g, 0.235 mol) were mixed in 350 ml of deionized water. After stirring for 30 min, 5-aminoisophthalic acid (5.303 g, 0.029 mol) was added into the mixture for further reaction. The resulting solution was kept at 80 °C with stirring for 30 h. After cooling to room temperature, the mixture was acidified with hydrochloric acid (2M) and centrifuged, and the white powder was obtained with the recrystallization from tetrahydrofuran (THF). ^1^H nuclear magnetic resonance (NMR) [400 MHz, dimethyl sulfoxide (DMSO)] δ 12.99 (s, 4H), 7.95 (d, *J* = 8.0 Hz, 4H), 7.79 (s, 1H), 7.47 to 7.34 (m, 6H), 4.94 (s, 4H); ^13^C NMR (101 MHz, DMSO) δ 167.64, 167.46, 148.48, 144.03, 132.44, 130.25, 130.03, 126.99, 118.49, 116.88, 54.85 (Figs. S15 and S16).

For synthesis of SOF-HTNI, a mixture solution of HT (0.045 g, 0.10 mmol) and NI (0.028 g, 0.10 mmol) in 10 ml of an ethanol–water mixture (v/v = 1:1) was ultrasonicated for 10 min at room temperature. The solution was then sealed in a 25-ml Teflon-lined reactor and heated to 150 °C for 48 h. After cooling to room temperature, yellow needle-shaped crystals were obtained with 51% yield based on the amount of the NI used. Analysis calcd. for C_44_H_35_N_3_O_8_ (%): C, 72.02; H, 4.81; N, 5.73; O, 17.44. Found (%): C, 72.16; H, 4.79; N, 6.15; O, 16.97. IR: 1696(m); 1602(vs); 1507(w); 1412(m); 1272(s); 1012(m); 834(m); 771(m); 680(w); 554(w).

### Iodine capture ability assay

For vapor-phase uptake capacity assay, 10 mg of SOF-HTNI was placed in a separate glass vial and sealed in an environment containing molecular iodine. The vial was heated to 75 °C, and the amount of iodine uptake was calculated using Eq. 1:

$$w=\frac{m_{t}-m_{0}}{m_{0}}$$ (1)

where *m_t_* and *m_0_* are the masses of compound before and after contact with iodine vapor, respectively.

For the uptake capacity in aqueous triiodide, the effect of pH on the capture of I_3_^−^ ions by SOF-HTNI was explored. I_3_^−^ solution was freshly prepared by mixing 12.7 mg of I_2_ and 16.6 mg of KI in 100 ml of aqueous solutions. The pH of the solutions was adjusted to final values of 3.0, 4.0, 5.0, 6.0, 7.0, 8.0, 9.0, and 10.0 using sodium hydroxide and hydrochloric acid. Then, 1 mg of SOF-HTNI was immersed in the freshly prepared solutions for 48 h. The initial and final concentrations of iodine were recorded by ultraviolet-visible (UV-vis) spectroscopy. The uptake capability of SOF-HTNI for I_3_^−^ was calculated by using Eq. 2:

$$q_{e}=\frac{\left(C_{0}-C_{e}\right)V}{m}$$ (2)

where *q_e_* (g g^−1^) is the equilibrium adsorption amount, *C_0_* and *C_e_* (mg l^−1^) are the concentrations of iodine before and after adsorption, *V* (l) is the volume of the solution, and *m* (mg) is the mass of the SOF-HTNI.

For adsorption kinetics of triiodide, the adsorption of iodine in aqueous solutions was examined by soaking 1 mg of the adsorbent in 150 ml of 0.3 mM I_3_^−^ solution, without adjusting the pH value of the solution. During the adsorption experiments, 3.0 ml of samples was removed and centrifuged at regular intervals for analysis. The adsorption kinetics were fitted with pseudo-first-order and pseudo-second-order models to investigate the adsorption mechanism by using Eqs. 3 and 4, respectively:

$$\text{ln}\left(q_{e}-q_{t}\right)=\text{ln}q_{e}-k_{1}t$$ (3)

$$\frac{t}{q_{t}}=\frac{1}{k_{2}{q_{e}}^{2}}+\frac{t}{q_{e}}$$ (4)

where *t* (h) is the contact time; *q_t_* (g g^−1^) and *q_e_* (g g^−1^) are the adsorption capacities of iodine at time *t* and the equilibrium time, respectively; and *k*_1_ and *k*_2_ (g g^−1^ h^−1^) are the rate constants.

For the adsorption isotherms of triiodide, the adsorptive isotherm was investigated in I_3_^−^ aqueous solution with different initial concentrations of I_3_^−^ (range, 0.5 to 8.0 mM). For this test, 1 mg of SOF-HTNI was dispersed into 10 ml of I_3_^−^ solutions with varying concentrations. The Langmuir and Freundlich adsorption isotherms were fitted with Eqs. 5 and 6, respectively:

$$\frac{C_{e}}{q_{e}}=\frac{C_{e}}{q_{m}}+\frac{1}{k_{3}q_{m}}$$ (5)

$$q_{e}=k_{4}C_{e}^{\frac{1}{n}}$$ (6)

where *C_e_* (g l^−1^) is the equilibrium concentration, *q_e_* (g g^−1^) is the adsorption capacity of I_3_^−^ at equilibrium, *q_m_* (g g^−1^) is the maximum amount of adsorption, and *k*_3_ and *k*_4_ (l g^−1^) are equilibrium constants.

For reversibility and stability assay of triiodide, 1 mg of SOF-HTNI was contacted with 10 ml of 2 mM I_3_^−^ solution. After 48 h, the solution was centrifuged, and the supernatant was analyzed by UV-vis spectroscopy to determine the adsorption capacity. The sediment was washed with methanol 3 times to remove the bound iodine. The adsorption–desorption process was then repeated to determine the reusability. The stability of used SOF-HTNI was analyzed by XRD.

For adsorption selectivity assay to triiodide, the ion selectivity was examined in 10 ml of 2 mM I_3_^−^-spiked aqueous solutions containing different interfering ions at equivalent concentrations with I_3_^−^, including Cl^−^, Br^−^, SO_4_^2−^, NO_3_^−^, and all of these anions combined. SOF-HTNI (1 mg) was added to the mixed solutions for 48 h, after which the solutions were centrifuged and the concentrations of I_3_^−^ in the liquid supernatant were measured. A blank test was conducted without the addition of the interfering ions. The adsorption experiment simulating the leakage of iodine pollution into seawater was carried out in 50 ppm of I^−^ ion-spiked natural seawater.

For the adsorption assays to iodide, 1 mg of SOF-HTNI was dispersed in 100 ml of I^−^ ion-spiked solution. The effect of pH on the capture of I^−^ ions by SOF-HTNI was investigated in a 16 ppm I^−^ aqueous solution across a pH range of 3 to 9. Additional adsorption tests for I^−^ ions were conducted in natural seawaters spiked with I^−^ ions. After continuous stirring for 12 h, aliquots of the dispersion were collected and filtered through a 0.45-μm membrane filter. The concentration of I^−^ ions in the filtrates was measured by the inductively coupled plasma mass spectrometry (ICP-MS) method, and the adsorption capacity was determined according to Eq. 2.

### Assay of adsorption capacity in seawater

Natural seawater was collected from the west coast of Haikou City, Hainan Province, China. Then, 10 mg of SOF-HTNI was placed into dialysis bags and kept in contact with 100 l of natural seawater for 20 d under stirring condition. The seawater was collected and filtered at regular intervals, and the concentrations in the seawater were determined by ICP-MS. The uptake capacities were calculated by using Eq. 2. The capacities to other metals were also determined by testing the contents of the adsorbent after adsorption.

## Data Availability

The data are available from the authors upon a reasonable request. The x-ray crystallographic coordinates for the structure have been deposited at the Cambridge Crystallographic Data Centre (CCDC), under deposition number 2164589. These data can be obtained free of charge from the CCDC via www.ccdc.cam.ac.uk.

## Supplementary Materials

Supplementary 1 Supplementary Methods Figs. S1 to S16 Tables S1 to S7 References
