# Supplementary material for: Supramolecular Organic Framework with Multidimensional Storage Spaces for Ultrahigh-Capacity Iodine Capture from Seawater
Source: Research (Wash D C). 2025 Feb 7;8:0608. doi: 10.34133/research.0608 (PMC12352584; doi:10.34133/research.0608)
Supplement: Supplementary 1 — Supplementary Methods Figs. S1 to S16 Tables S1 to S7 References [file research.0608.f1.docx]

Supplementary Materials for

**Supramolecular Organic Framework with Multi-Dimensional Storage Spaces for Ultrahigh-Capacity Iodine Capture from Seawater**

Lijuan Feng^1†^, Jun Zhang^1†^, Jiacheng Zhang^1^, Xuewen Cao^1^, Yihui Yuan^1^*, Ning Wang^1^*

^1^State Key Laboratory of Marine Resource Utilization in South China Sea, Collaborative Innovation Center of Marine Science and Technology, Hainan University, Haikou, 570228, P. R. China

*Address correspondence to: Ning Wang; wangn02@foxmail.com and Yihui Yuan; [yuanyh@hainanu.edu.cn](mailto:yuanyh@hainanu.edu.cn)

^†^These authors contributed equally to this work.

**Supplementary Methods**

**Material characterization.** PXRD data were recorded on a Rigaku Smart Lab X-ray diffractometer with Cu-Kα radiation (λ = 1.5418 Å) at 50 kV and 20 mA, with a step size of 0.01° and a scanning rate of 10° min^−1^. The simulated powder pattern was generated using Mercury software. The Fourier transform infrared (FT-IR) spectrum was measured on PerkinElmer Frontier from 500 to 4000 cm^−1^ with KBr pellets. Elemental analyses were conducted on a Vario EL cube instrument. TGA was performed from room temperature to 800 °C at a heating rate of 10 °C min^−1^ under N_2_ atmosphere. The N_2_ adsorption/desorption isotherm was measured at 77 K by a Micromeritics ASAP 2460 instrument. Prior to test, the SOF was degassed for 12 h at 150 °C under high vacuum. The water contact angle was measured by a JC2000D4 contact angle system at room temperature with the powder tableted material. Raman spectra were obtained on a Renishaw inVia Raman spectrophotometer. Fluorescence analysis was carried out on a FS5 Spectrofluorometer from Edinburgh Instruments at room temperature. The ligand HT and NI samples were scanned with excitation wavelengths of 357 nm and 390 nm, respectively. ESR data were recorded using a Bruker A300-12 spectrometer. XPS spectra were obtained using Thermo Escalab 250Xi equipment. ICP-MS was conducted on an Agilent ICPMS7899 instrument.

**X-ray crystallography.** Single-crystal X-ray diffraction data were collected on a Rigaku Oxford diffractometer with Cu Kα radiation (λ = 1.54178 Å) at 150 K. The structure was solved by direct methods using SHELXL-2014 and refined by the ShelXL refinement package using Least Squares minimization.

**Computer calculations.**

Density functional theory (DFT) calculations were performed using Vienna Ab-initio Simulation Package (VASP). The system was set up, run, and analyzed through a complete graphical interface for VASP calculations. The projected augmented wave (PAW) potential was used to describe the core electrons, and the calculations were performed within the framework of the generalized gradient approximation (GGA), specifically using the Perdew-Burke-Ernzerh (PBE) functional to calculate the exchange-correlation energy. The plane wave basis set was extended with a cut-off energy of 450 eV. A 2×2×2 Monkhorst Pack k-point grid was used for structure optimizations and electronic structure calculations. The conjugate gradient method was employed to fully optimize the positions of the system until all forces on each atom were reduced to less than 0.02 eV/Å. The energy convergence of the self-consistent process was set to 10^-5^ eV. Gaussian smearing broadening was set to 0.05 eV. Electron surface potential (ESP) and Charge density difference (CDD) was visualized with VESTA.

**Supplementary Figures**


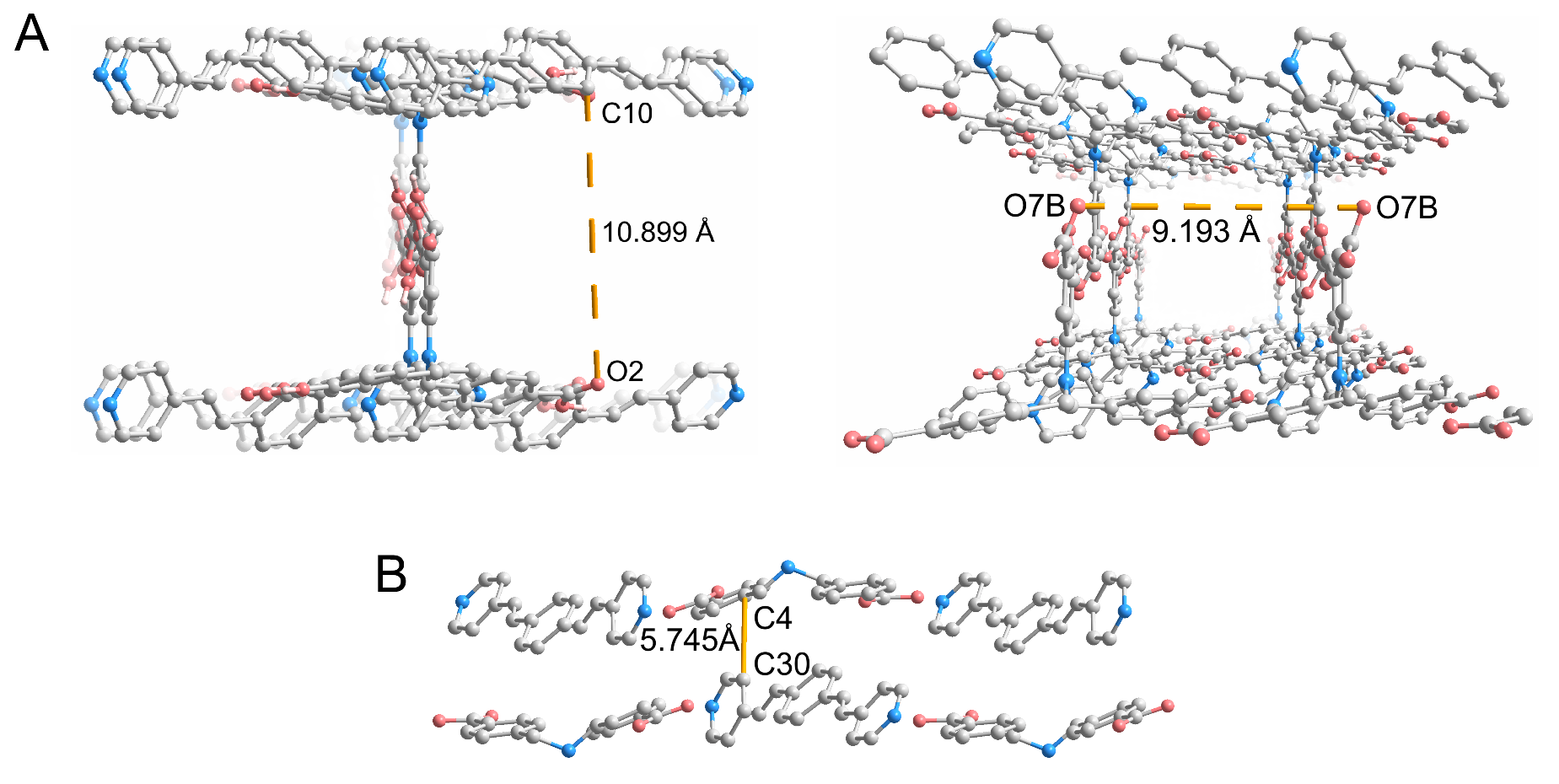


**Fig. S1** The measured distances of the 1D channel (A) and 2D interlayer (B) space for SOF-HTNI. The perpendicular and adjacent O2-C10, O7B-O7B, and C30-C4 atoms were selected.


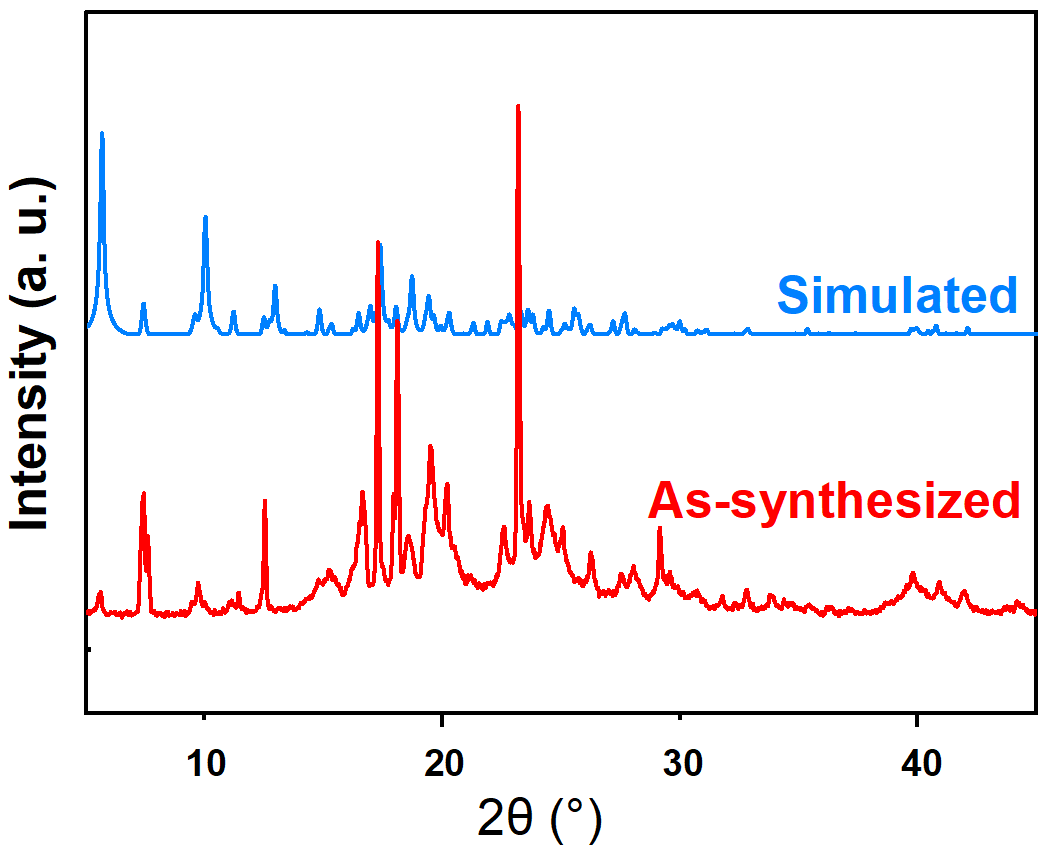


**Fig. S2.** PXRD patterns of the as-synthesized sample and the simulated data.


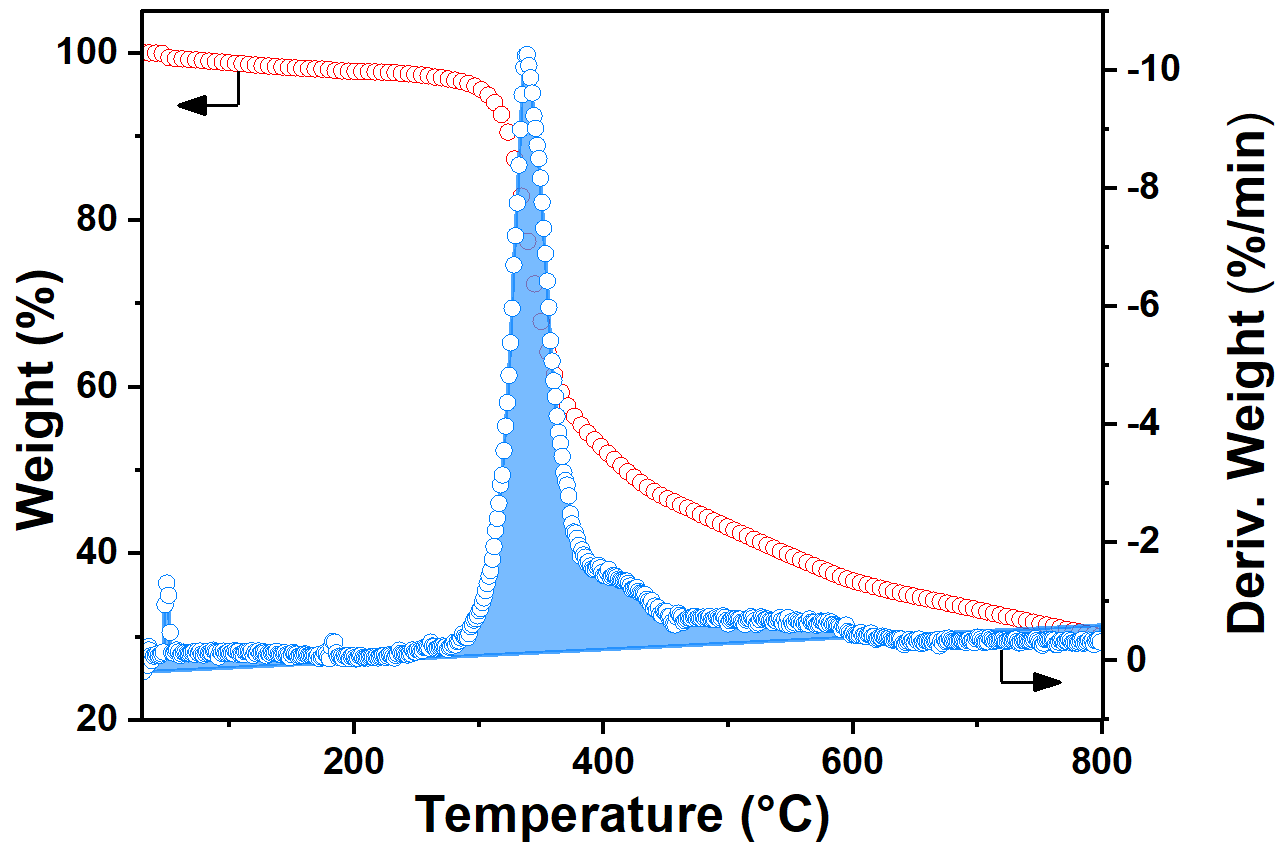


**Fig. S3.** TG-DTG analysis of SOF-HTNI.


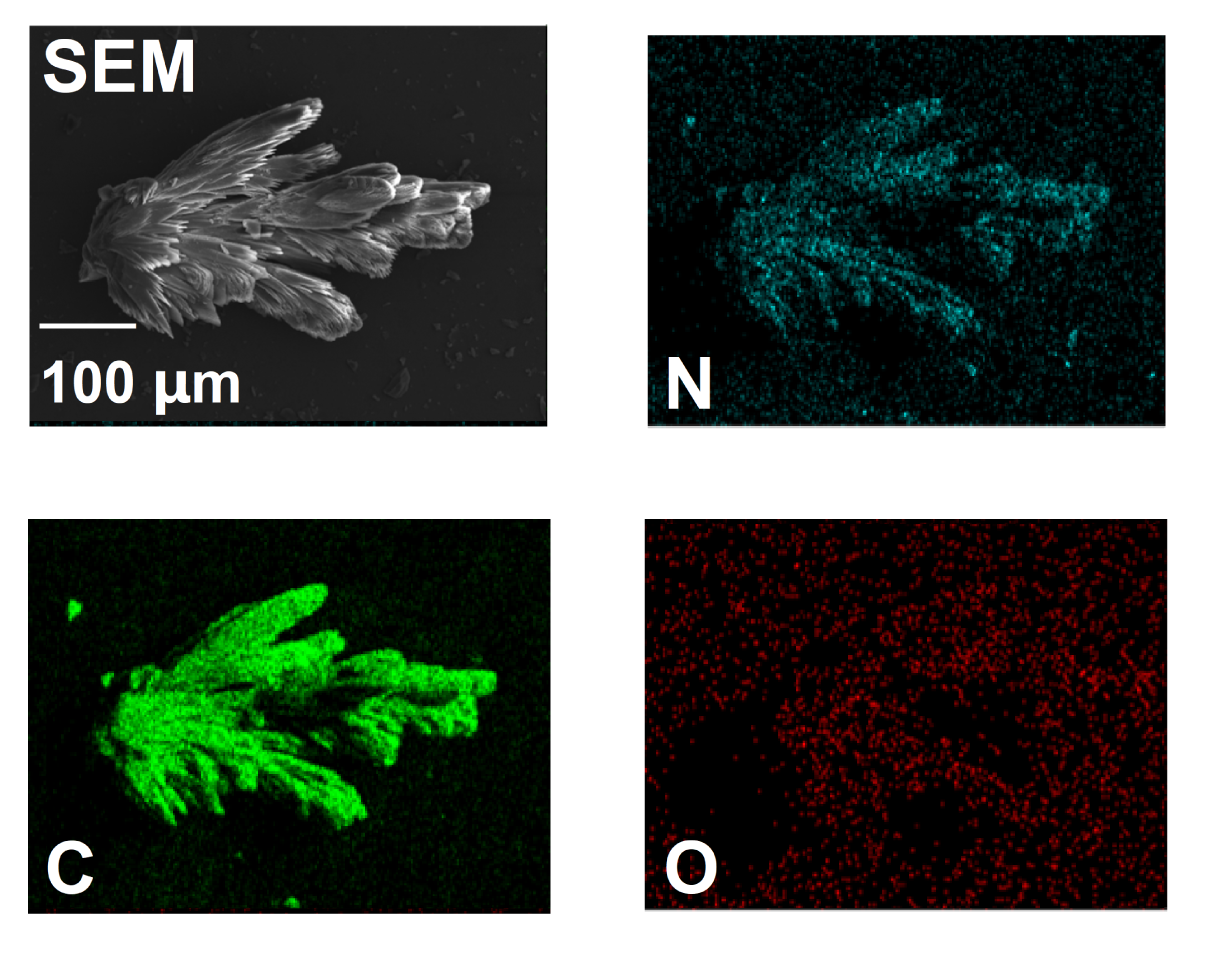


**Fig. S4.** SEM image and EDS mapping of SOF-HTNI.


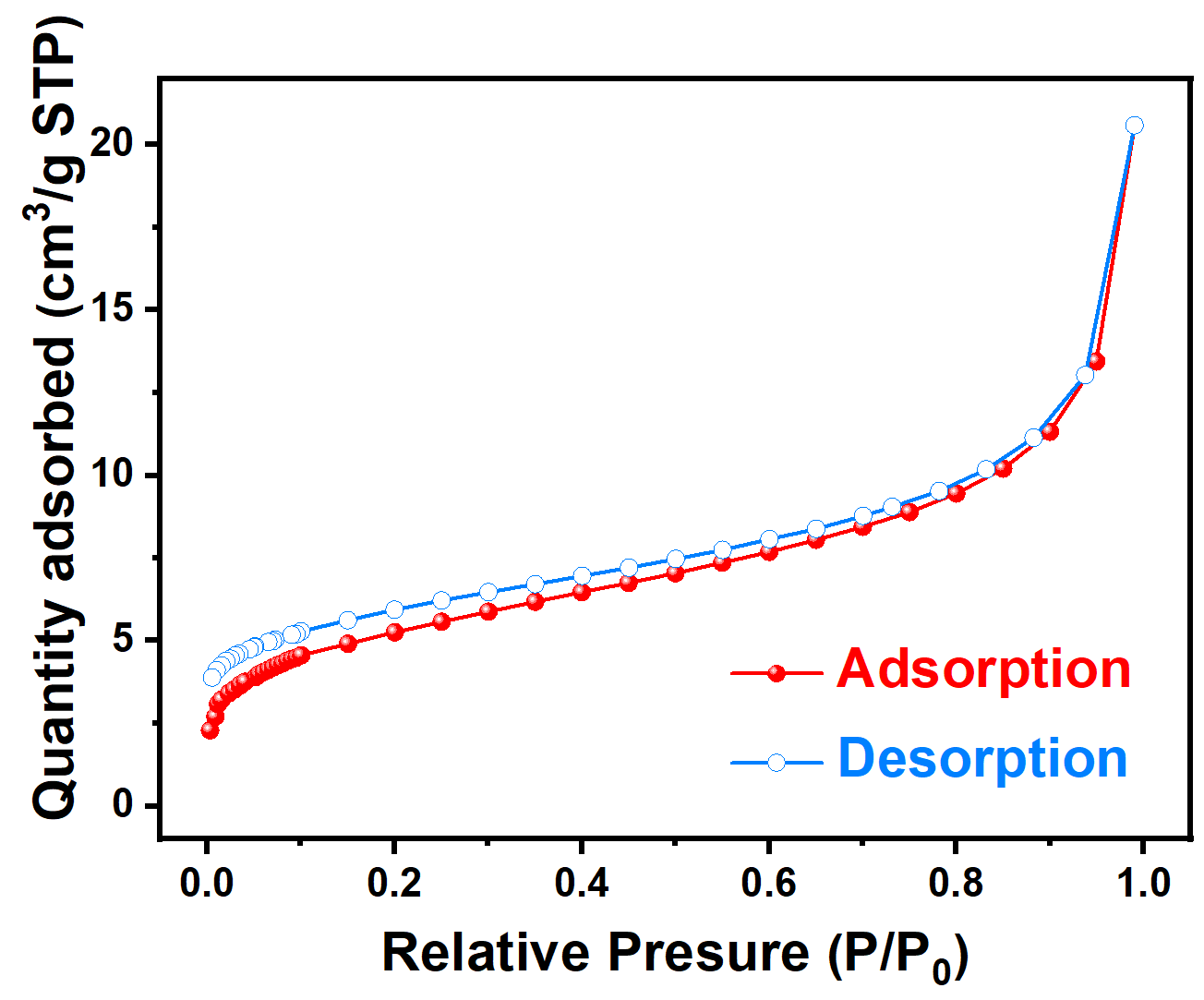


**Fig. S5.** N_2_ adsorption/desorption isotherms of SOF-HTNI.


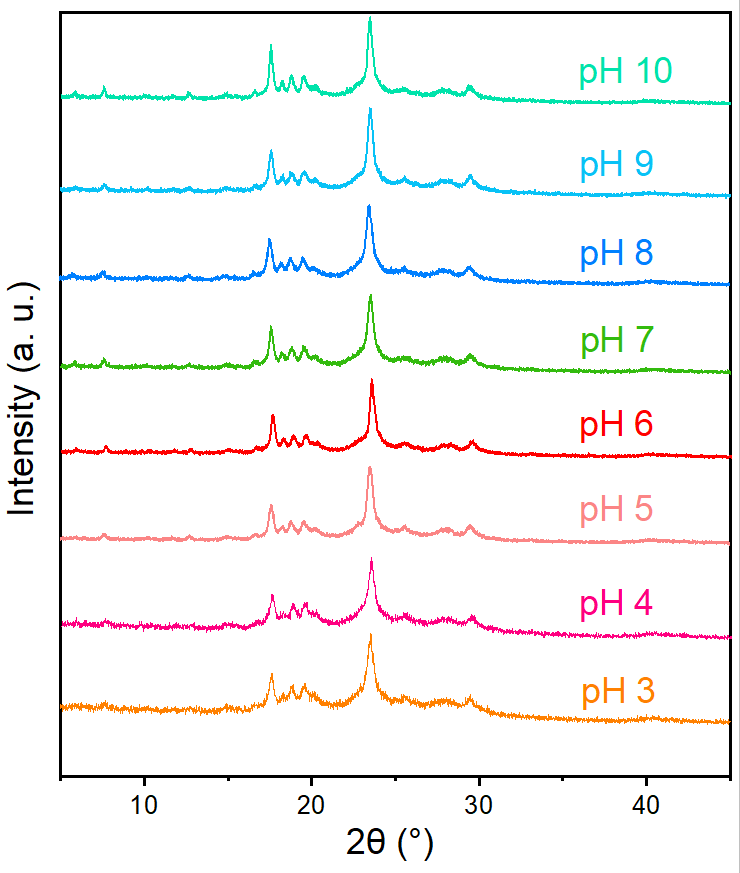


**Fig. S6.** PXRD patterns of SOF-HTNI treated with different pH for 2 days.


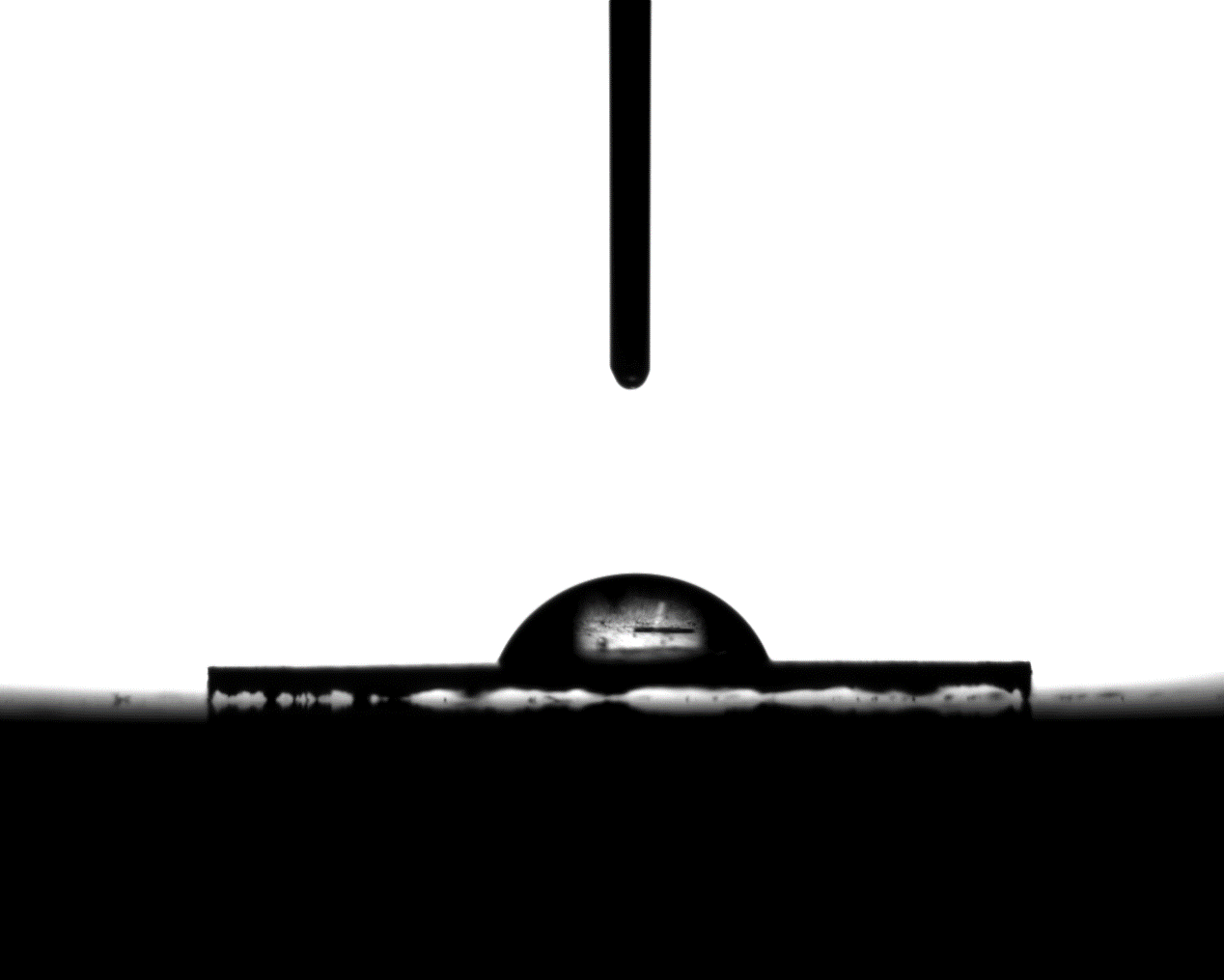


**Fig. S7.** Water contact angles of SOF-HTNI.

**
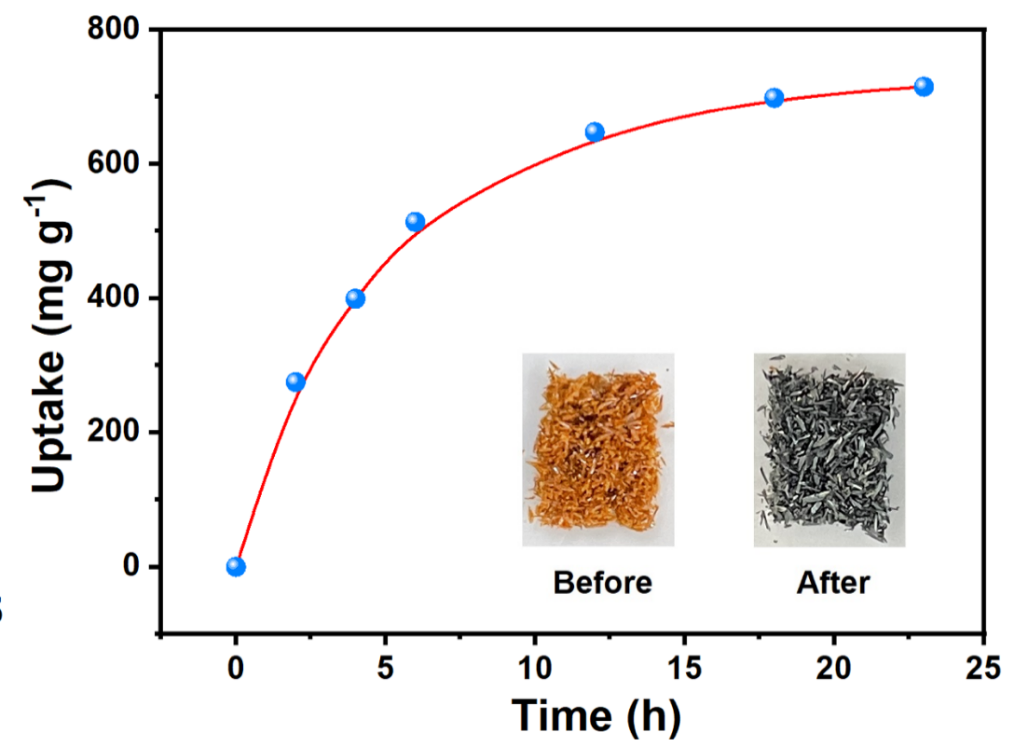
**

**Fig. S8.** Iodine uptake capacity of SOF-HTNI in the iodine vapor phase.


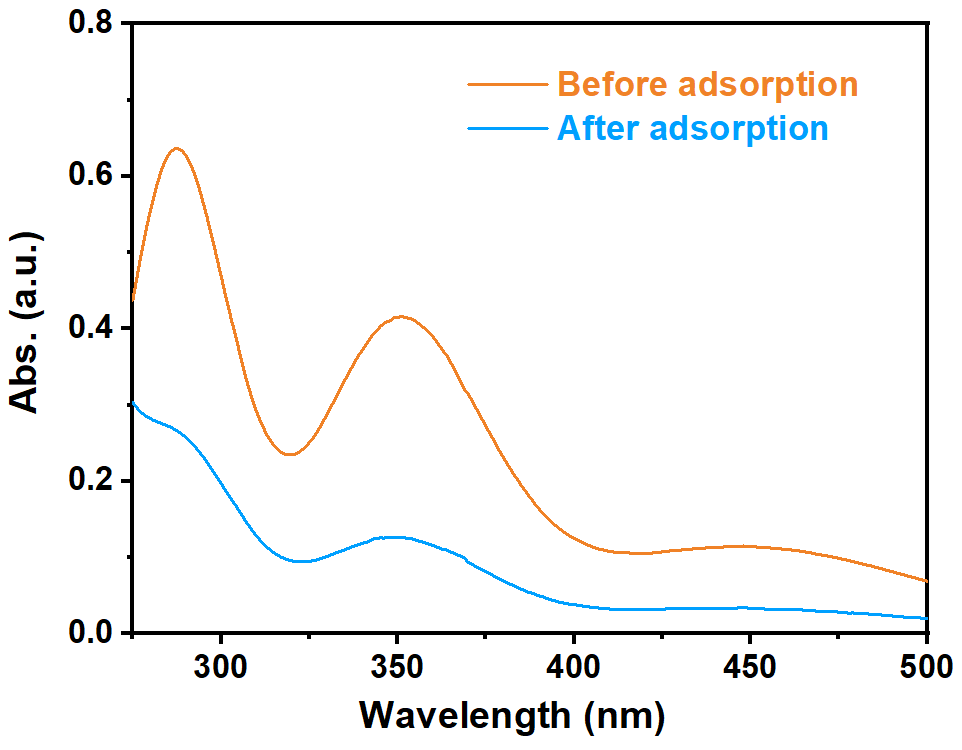


**Fig. S9.** The UV spectra before and after triiodide uptake by SOF-HTNI in the aqueous phase.


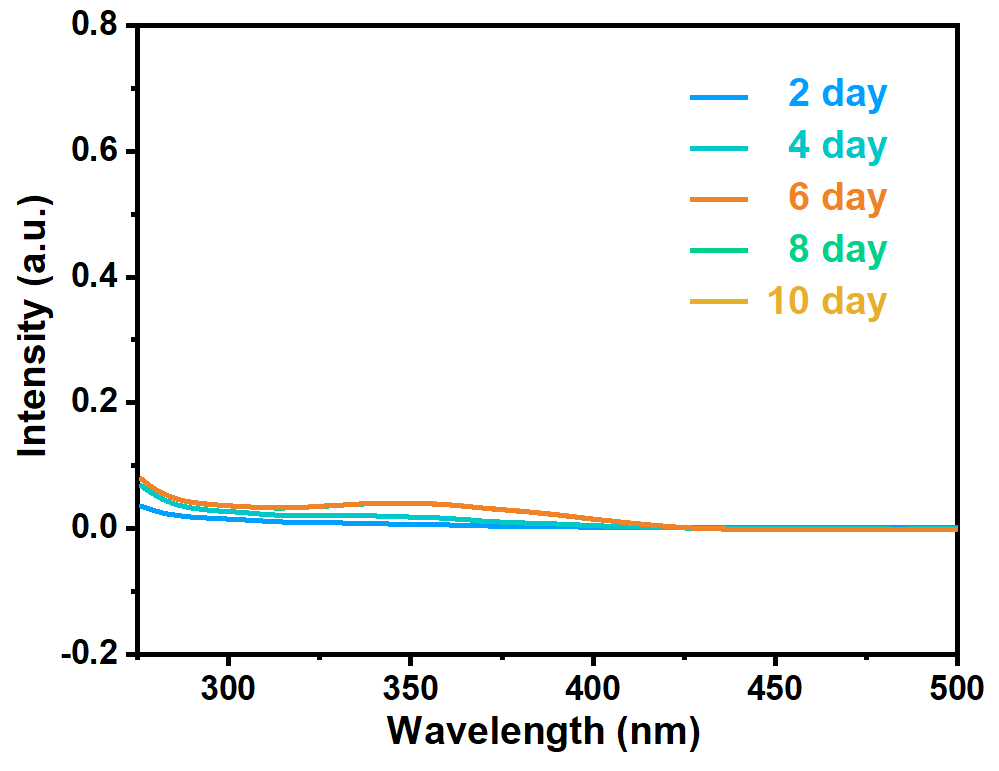


**Fig. S10.** Leakage of I_3_^−^ from I_3_^−^ ion-loaded SOF-HTNI during a 10-day soaking in pure water at room temperature.


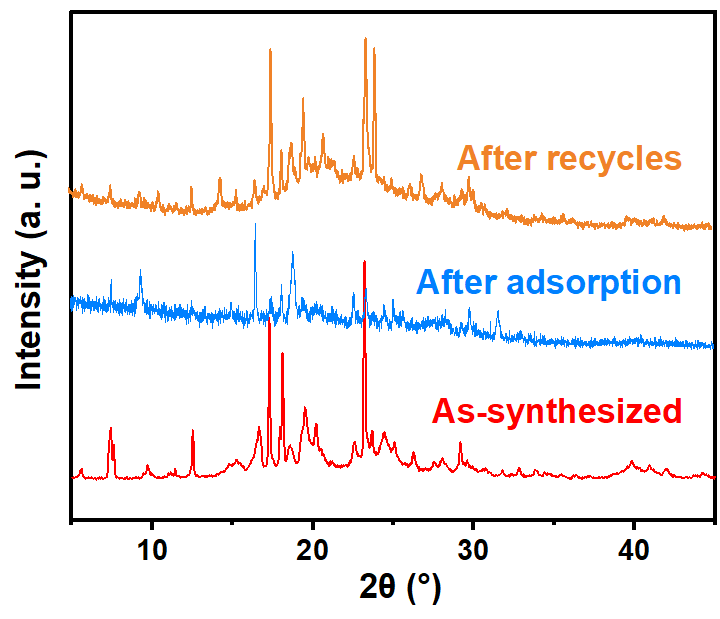


**Fig. S11.** PXRD patterns of SOF-HTNI after adsorption and recycles for I_3_^−^.


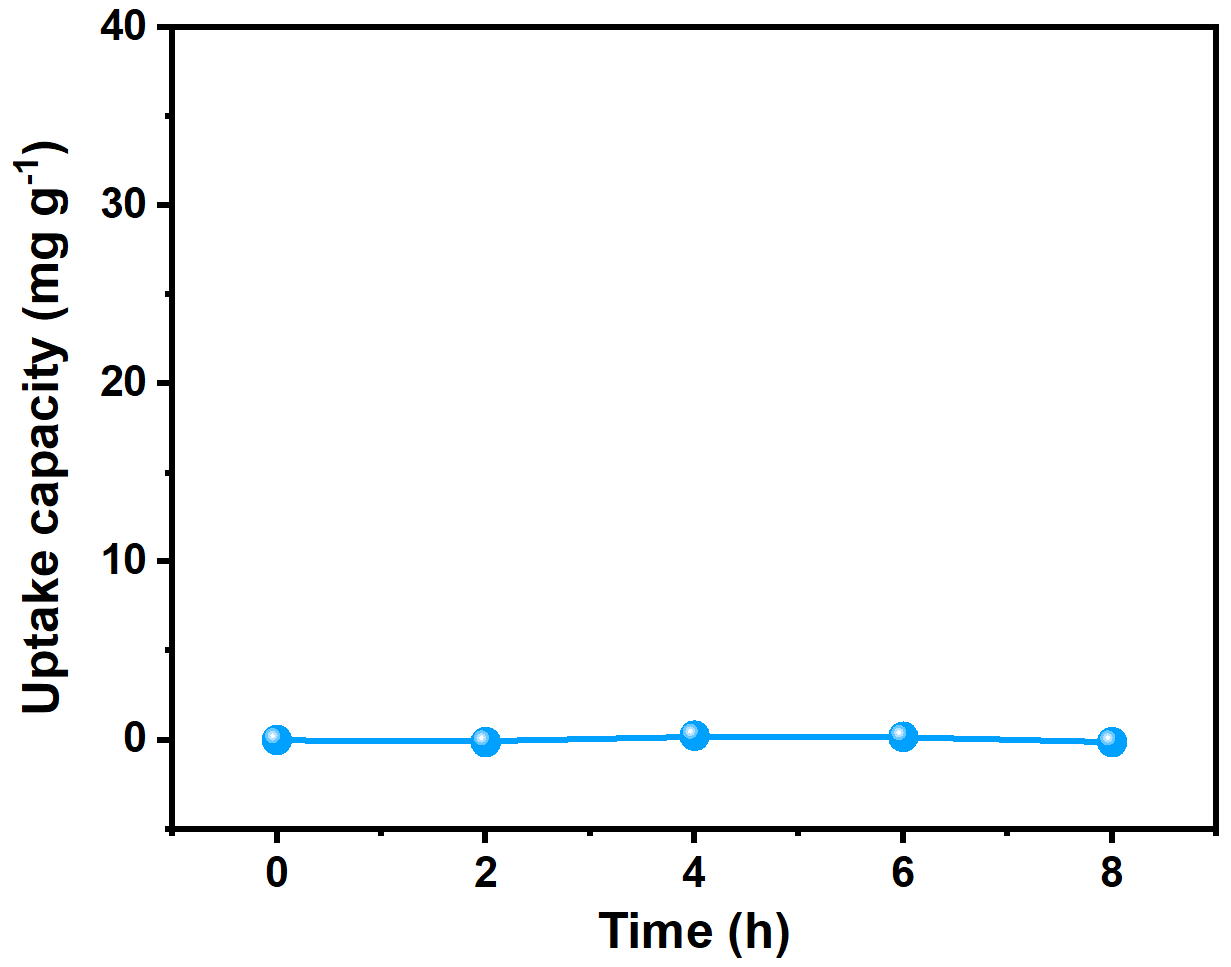


**Fig. S12.** Adsorption kinetics of SOF-HTNI for IO_3_^−^ ions. The concentration of IO_3_^−^ ions is 16 ppm based on the iodine content, and the dosage of SOF-HTNI is 10 mg L^−1^.


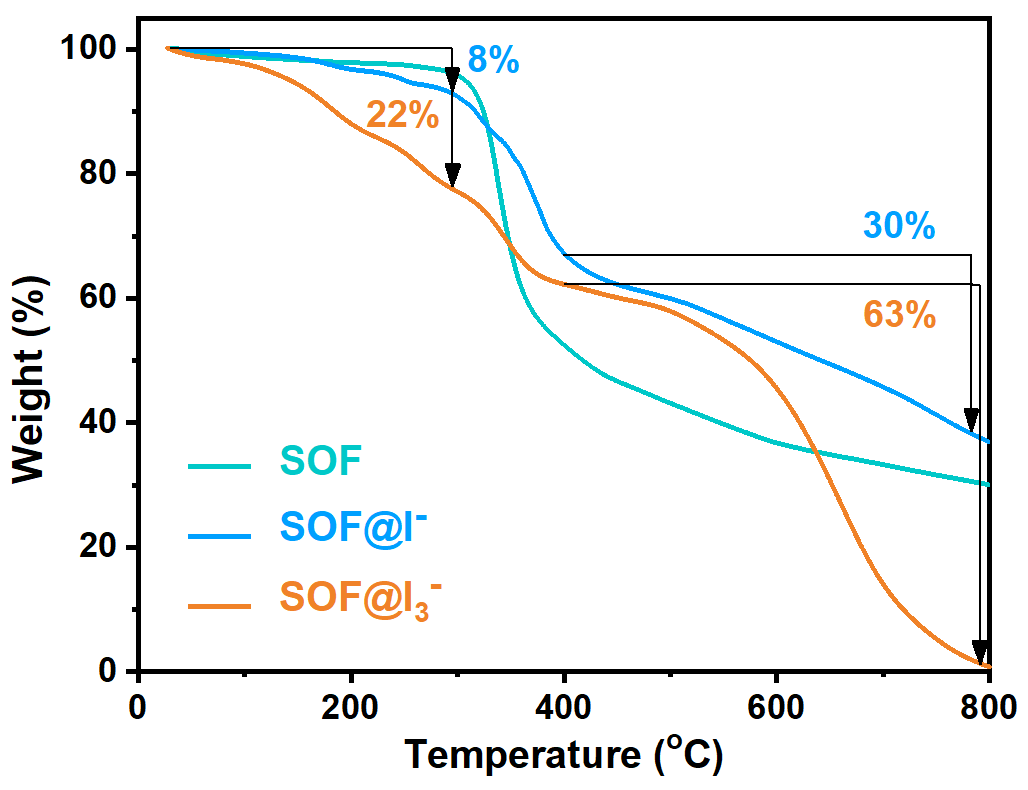


**Fig. S13.** TG analysis of SOF-HTNI before and after the adsorption of I^−^ and I_3_^−^ ions.


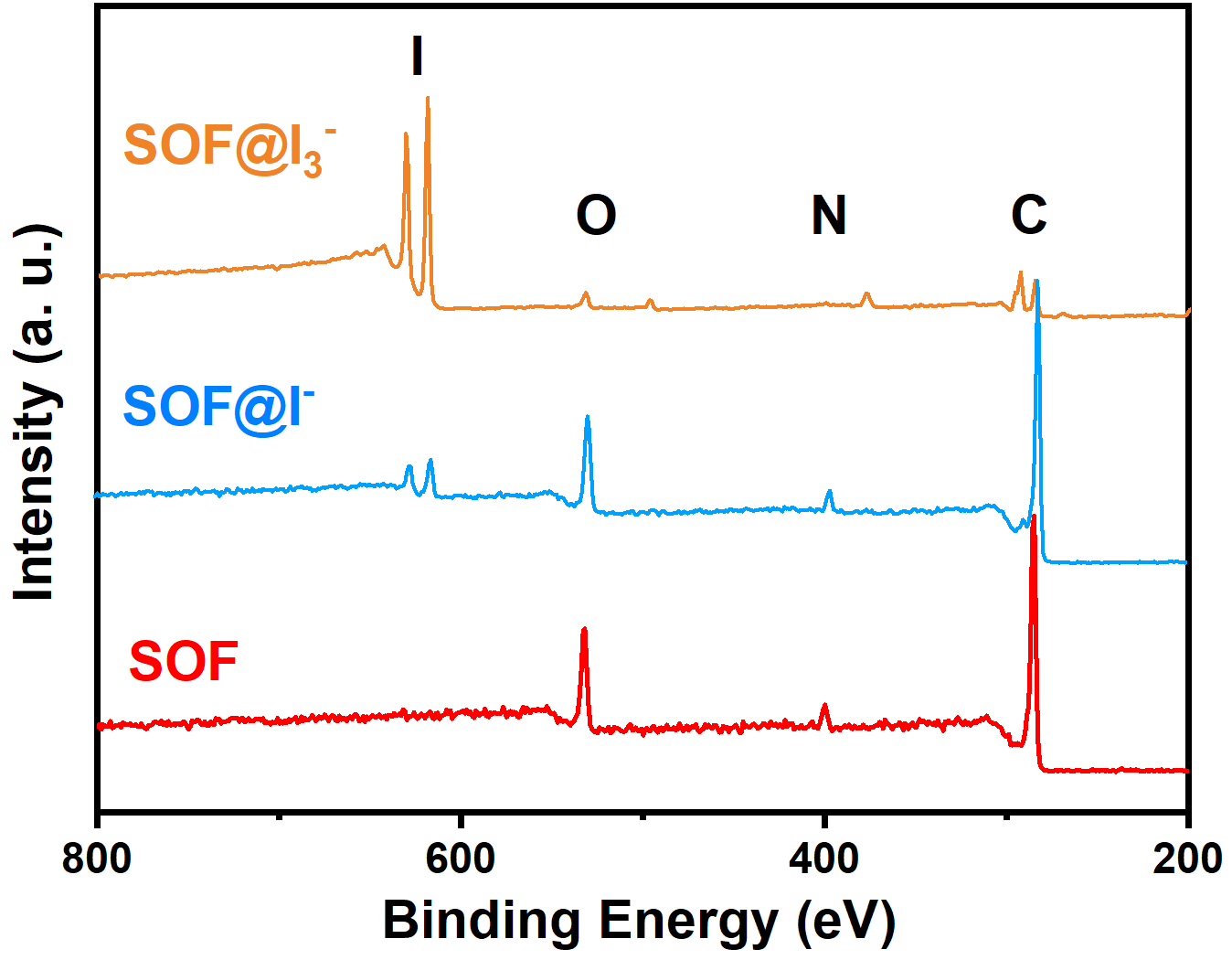


**Fig. S14.** XPS spectra of SOF-HTNI before and after adsorption I^−^ and I_3_^−^ ions in aqueous solution.


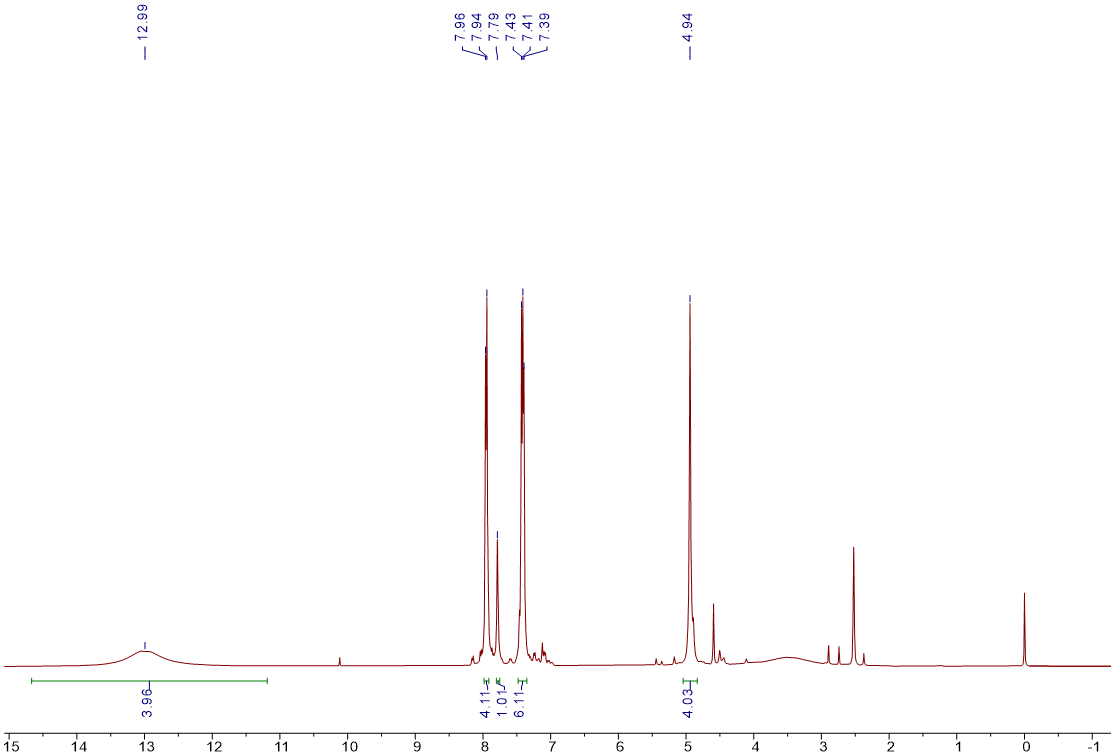


**Fig. S15.** ^1^H NMR spectra of HT in DMSO.


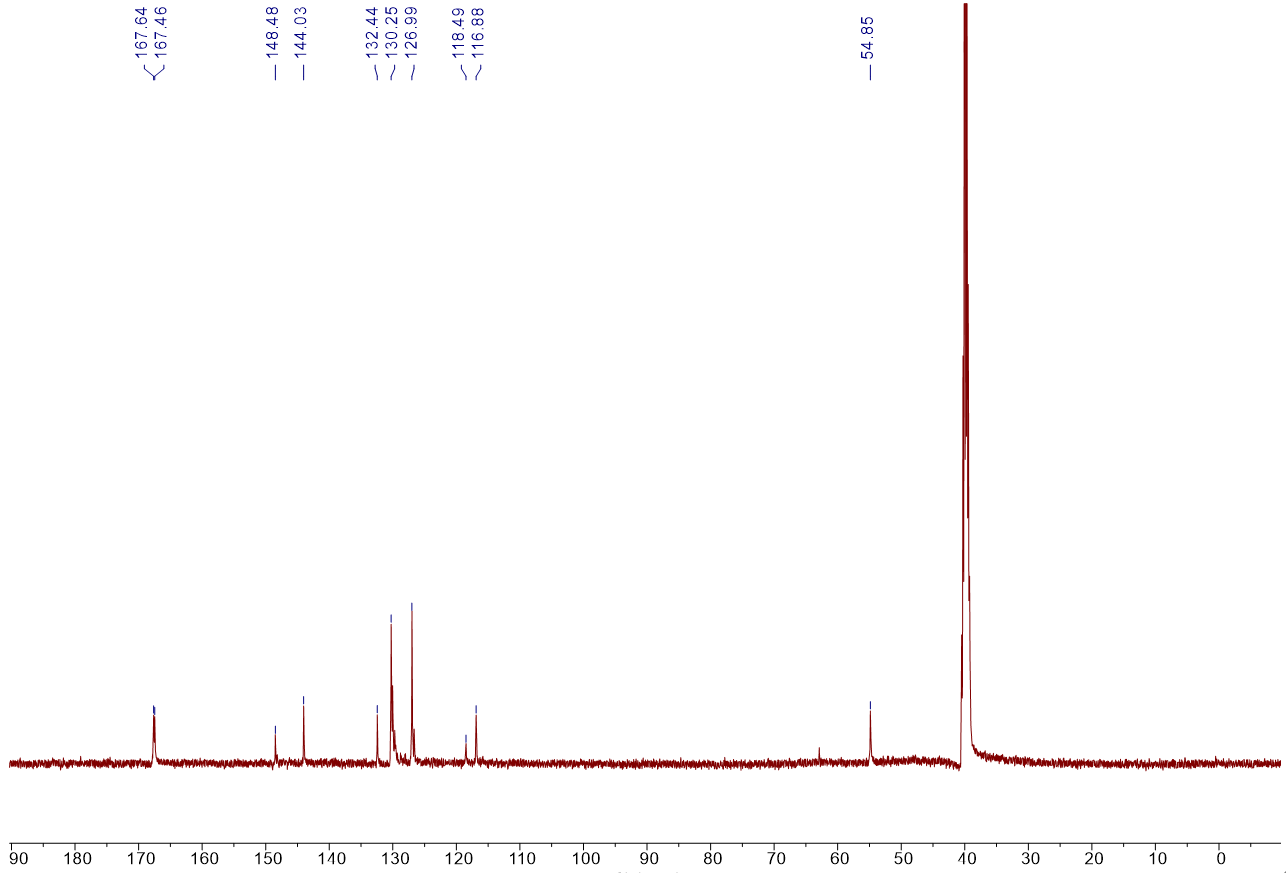


**Fig. S16.** ^13^C NMR spectra of HT in DMSO.

**Supplementary Tables**

**Table S1.** Crystallographic data for SOF-HTNI.

|  | SOF-HTNI |
| --- | --- |
| Empirical formula | C_44_H_35_N_3_O_8_ |
| Formula weight | 733.75 |
| Crystal system | monoclinic |
| Space group | *P2_1_/c* |
| Unit cell dimensions |  |
| a (Å) | 9.1930(2) |
| b (Å) | 30.4624(9) |
| c (Å) | 18.5161(4) |
| α (°) | 90.00 |
| β (°) | 97.972(2) |
| γ (°) | 90.00 |
| V (Å^3^) | 5135.2 (2) |
| Z | 4 |
| Dcalc (g cm^-3^) | 0.949 |
| F (000) | 1536.0 |
| Crystal size (mm) | 0.12×0.1×0.1 |
| Limiting indices | -11≤h≤9  -32≤k≤37  -22≤l≤22 |
| Reflection collected | 0.1196 |
| Independent reflections | 1061 |
| Goodness-of-fit on F^2^ | 1.019 |
| R (reflections) | 0.1196 |
| wR2 (reflections) | 0.2928 |

**Table S2.** Bond lengths [Å] for SOF-HTNI.

| **Atom** | **Atom** | **Length/Å** |  | **Atom** | **Atom** | **Length/Å** |
| --- | --- | --- | --- | --- | --- | --- |
| O3 | C21 | 1.201(6) |  | C41 | C43 | 1.513(8) |
| O1 | C36 | 1.302(6) |  | C25 | C24 | 1.346(6) |
| O4 | C21 | 1.308(6) |  | C25 | C28 | 1.533(5) |
| O5 | C44 | 1.267(7) |  | C24 | C23 | 1.405(5) |
| O8 | C43 | 1.251(8) |  | C44 | O6A | 1.178(11) |
| O2 | C36 | 1.188(6) |  | C43 | O7A | 1.29(3) |
| O7B | C43 | 1.254(10) |  | N1 | C5 | 1.313(7) |
| O6B | C44 | 1.43(3) |  | N1 | C1 | 1.317(7) |
| N3 | C37 | 1.370(6) |  | N2 | C19 | 1.318(8) |
| N3 | C29 | 1.450(5) |  | N2 | C18 | 1.283(8) |
| N3 | C28 | 1.462(5) |  | C5 | C4 | 1.377(6) |
| C33 | C34 | 1.362(6) |  | C7 | C6 | 1.264(7) |
| C33 | C32 | 1.346(6) |  | C7 | C8 | 1.471(6) |
| C33 | C36 | 1.529(5) |  | C6 | C3 | 1.450(6) |
| C37 | C38 | 1.412(5) |  | C3 | C4 | 1.394(7) |
| C37 | C42 | 1.401(6) |  | C3 | C2 | 1.397(7) |
| C29 | C30 | 1.514(5) |  | C11 | C14 | 1.465(6) |
| C39 | C38 | 1.403(7) |  | C11 | C12 | 1.333(8) |
| C39 | C44 | 1.525(7) |  | C11 | C10 | 1.399(8) |
| C39 | C40 | 1.349(7) |  | C14 | C15 | 1.140(9) |
| C30 | C31 | 1.365(6) |  | C12 | C13 | 1.397(7) |
| C30 | C35 | 1.353(6) |  | C20 | C16 | 1.356(9) |
| C21 | C22 | 1.507(6) |  | C20 | C19 | 1.405(8) |
| C26 | C27 | 1.401(6) |  | C13 | C8 | 1.379(7) |
| C26 | C25 | 1.376(6) |  | C2 | C1 | 1.373(7) |
| C34 | C35 | 1.396(5) |  | C8 | C9 | 1.387(8) |
| C31 | C32 | 1.399(6) |  | C15 | C16 | 1.476(7) |
| C22 | C27 | 1.348(6) |  | C16 | C17 | 1.387(10) |
| C22 | C23 | 1.378(6) |  | C9 | C10 | 1.407(7) |
| C41 | C42 | 1.378(7) |  | C18 | C17 | 1.371(8) |
| C41 | C40 | 1.388(7) |  |  |  |  |

**Table S3.** Bond angles [^o^] for SOF-HTNI.

| **Atom** | **Atom** | **Atom** | **Angle/˚** | **Atom** | **Atom** | **Atom** | **Angle/˚** |
| --- | --- | --- | --- | --- | --- | --- | --- |
| C37 | N3 | C29 | 122.4(3) | O5 | C44 | O6B | 120.3(11) |
| C37 | N3 | C28 | 120.0(4) | O5 | C44 | C39 | 115.9(6) |
| C29 | N3 | C28 | 117.2(4) | O6B | C44 | C39 | 112.1(10) |
| C34 | C33 | C36 | 117.0(4) | O6A | C44 | O5 | 122.5(7) |
| C32 | C33 | C34 | 119.5(4) | O6A | C44 | C39 | 120.4(7) |
| C32 | C33 | C36 | 123.4(4) | C39 | C40 | C41 | 121.7(5) |
| N3 | C37 | C38 | 121.1(4) | O8 | C43 | O7B | 125.9(7) |
| N3 | C37 | C42 | 121.3(4) | O8 | C43 | C41 | 117.0(6) |
| C42 | C37 | C38 | 117.7(5) | O8 | C43 | O7A | 110.6(14) |
| N3 | C29 | C30 | 115.4(4) | O7B | C43 | C41 | 116.6(7) |
| C38 | C39 | C44 | 120.4(5) | O7A | C43 | C41 | 114.6(12) |
| C40 | C39 | C38 | 120.0(5) | C5 | N1 | C1 | 117.0(4) |
| C40 | C39 | C44 | 119.6(6) | C18 | N2 | C19 | 116.2(5) |
| C31 | C30 | C29 | 120.0(4) | N1 | C5 | C4 | 123.4(5) |
| C35 | C30 | C29 | 121.8(4) | C6 | C7 | C8 | 128.2(5) |
| C35 | C30 | C31 | 118.2(4) | C7 | C6 | C3 | 126.7(6) |
| O3 | C21 | O4 | 124.4(4) | C4 | C3 | C6 | 124.0(5) |
| O3 | C21 | C22 | 122.7(5) | C4 | C3 | C2 | 115.9(4) |
| O4 | C21 | C22 | 112.8(5) | C2 | C3 | C6 | 120.1(5) |
| C25 | C26 | C27 | 119.7(4) | C12 | C11 | C14 | 118.2(6) |
| C33 | C34 | C35 | 120.2(4) | C12 | C11 | C10 | 118.8(5) |
| C30 | C31 | C32 | 121.2(5) | C10 | C11 | C14 | 122.9(6) |
| C39 | C38 | C37 | 120.0(4) | C15 | C14 | C11 | 136.4(9) |
| C27 | C22 | C21 | 122.2(4) | C11 | C12 | C13 | 122.1(6) |
| C27 | C22 | C23 | 119.7(4) | C16 | C20 | C19 | 121.2(7) |
| C23 | C22 | C21 | 118.0(4) | C5 | C4 | C3 | 120.1(5) |
| C22 | C27 | C26 | 120.7(4) | C8 | C13 | C12 | 121.2(6) |
| C42 | C41 | C40 | 118.8(5) | C1 | C2 | C3 | 118.8(6) |
| C42 | C41 | C43 | 118.5(5) | C13 | C8 | C7 | 120.8(5) |
| C40 | C41 | C43 | 122.7(6) | C13 | C8 | C9 | 116.9(4) |
| C26 | C25 | C28 | 118.7(4) | C9 | C8 | C7 | 122.3(5) |
| C24 | C25 | C26 | 119.7(4) | C14 | C15 | C16 | 134.1(10) |
| C24 | C25 | C28 | 121.7(4) | C20 | C16 | C15 | 126.0(8) |
| C30 | C35 | C34 | 120.8(4) | C20 | C16 | C17 | 115.5(5) |
| C33 | C32 | C31 | 119.8(4) | C17 | C16 | C15 | 118.1(8) |
| C25 | C24 | C23 | 120.7(4) | N2 | C19 | C20 | 121.8(7) |
| N3 | C28 | C25 | 114.1(3) | N1 | C1 | C2 | 124.8(5) |
| C22 | C23 | C24 | 119.5(4) | C8 | C9 | C10 | 121.6(5) |
| C41 | C42 | C37 | 121.7(4) | C11 | C10 | C9 | 119.3(6) |
| O1 | C36 | C33 | 111.1(4) | N2 | C18 | C17 | 126.6(8) |
| O2 | C36 | O1 | 125.2(4) | C18 | C17 | C16 | 118.4(7) |
| O2 | C36 | C33 | 123.6(4) |  |  |  |  |

**Table S4.** Hydrogen Bond lengths [Å] and angles [°] for SOF-HTNI.

| D | H | A | d(D-H)/Å | d(H-A)/Å | d(D-A)/Å | D-H-A/° |
| --- | --- | --- | --- | --- | --- | --- |
| O1 | H1 | N1 | 0.84 | 1.80 | 2.630(5) | 169.8 |
| O4 | H4 | N2 | 0.84 | 1.82 | 2.637(5) | 163.2 |
| O5 | H5 | O8 | 0.84 | 1.67 | 2.505(5) | 175.7 |

**Table S5.** Comparison of iodine uptake performance with currently available noncovalent organic framework materials in aqueous solution.

| Materials |  | solutions | | Vapor | | Ref. S |
| --- | --- | --- | --- | --- | --- | --- |
|  | Functional sites | Time | Q_e_(g g^−1^) | Time | Q_e_(g g^−1^) |  |
| SOF-HTNI | amine/pyridine | 29 h | 4.83 | 23 h | 0.715 | This work |
| Cu12b-NACs | Cyclic molecular | 60 min | 2.96 | / | / | 1 |
| HOF_T-Hex | amide | 40 min | 3.6 | 3 h | 6.4 | 2 |
| H_C_OFs | guest molecules | 24 h | 2.1 | 4 h | 2.9 | 3 |
| H_C_OF-7 |  | 6h | 1.39 | / | / | 4 |
| TIEPE-DABCO | Nitrogen Heterocycle | 5 h | 1.8 | 15 h | 3.23 | 5 |
| G-TP5 | Cyclic molecular | 60 min | 0.244 | 20 h | 0.67 | 6 |
| JLUE-SOF-3-DMSO | hydrazide/pyridine | 33 h | 0.207 | / | / | 7 |
| HOF-TAM–BPY | pyridine | 200 min | 1.12 | 15 h | 7.83 | 8 |
| Q[8]-(4-AP) | cucurbit[n]uril | 5 min | 0.120 | 250 min | 1.74 | 9 |
| MSOF | Ag | 6 h | 0.169 | 10 h | 3.62 | 10 |
| MPM-1-TIFSIX | Adenine | 96 h | 0.205 | / | / | 11 |

**Table S6.** I^−^ ions concentrations in the solutions during the adsorption process.

|  |  | Concentration* (ppb) | | | | q_e_ (mg g^-1^) | | |
| --- | --- | --- | --- | --- | --- | --- | --- | --- |
|  |  | Initial | Rep 1 | Rep 2 | Rep 3 | Rep 1 | Rep 2 | Rep 3 |
| pH | 3 | 3555.81 | 3466.096 | 3432.382 | 3347.428 | 44.857 | 61.714 | 104.191 |
|  | 4 | 3454.417 | 3321.847 | 3285.103 | 3230.305 | 66.285 | 84.657 | 112.056 |
|  | 5 | 3582.874 | 3393.54 | 3315.65 | 3292.87 | 94.667 | 133.612 | 145.002 |
|  | 6 | 3229.656 | 2824.418 | 2771.848 | 2742.798 | 202.619 | 228.904 | 243.429 |
|  | 7 | 3649.038 | 3027.42 | 2981.134 | 3009.608 | 310.809 | 333.952 | 319.715 |
|  | 8 | 3875.741 | 3061.647 | 3012.503 | 3011.839 | 407.047 | 431.619 | 431.951 |
|  | 9 | 3792.361 | 2767.601 | 2762.171 | 2815.199 | 512.38 | 515.095 | 488.581 |
| Time (h) | 1 | 3551.378 | 3438.428 | 3376.336 | 3324.282 | 56.475 | 87.521 | 113.548 |
|  | 2 | 3551.378 | 3179.632 | 3112.37 | 3183.726 | 185.873 | 219.504 | 183.826 |
|  | 4 | 3551.378 | 3015.888 | 3077.706 | 3054.27 | 267.745 | 236.836 | 248.554 |
|  | 6 | 3551.378 | 2841.948 | 2927.122 | 2951.096 | 354.715 | 312.128 | 300.141 |
|  | 8 | 3551.378 | 2801.214 | 2814.428 | 2863.57 | 375.082 | 368.475 | 343.904 |
|  | 10 | 3551.378 | 2683.99 | 2686.304 | 2753.262 | 433.694 | 432.537 | 399.058 |
|  | 12 | 3551.378 | 2656.748 | 2669.332 | 2708.64 | 447.315 | 441.023 | 421.369 |
| C_0_ (ppm) | 2 | 477.466 | 423.78 | 430.362 | 384.756 | 26.843 | 23.552 | 46.355 |
|  | 4 | 819.987 | 514.179 | 491.117 | 439.249 | 152.904 | 164.435 | 190.369 |
|  | 8 | 1639.391 | 970.109 | 974.535 | 864.727 | 334.641 | 332.428 | 387.332 |
|  | 16 | 3368.746 | 2483.056 | 2522.726 | 2615.418 | 442.845 | 423.01 | 376.664 |
|  | 32 | 6434.6 | 5507.744 | 5529.458 | 5576.42 | 463.428 | 452.571 | 429.09 |
| Ions | I^-^ | 3568.048 | 2711.098 | 2672.644 | 2701.042 | 428.475 | 447.702 | 433.503 |
|  | I^-^/Cl^-^ | 3349.854 | 2465.09 | 2497.788 | 2515.63 | 442.382 | 426.033 | 417.112 |
|  | I^-^/Br^-^ | 3444.818 | 2608.722 | 2555.392 | 2633.906 | 418.048 | 444.713 | 405.456 |
|  | I^-^/SO_4_^2-^ | 3542.259 | 2687.689 | 2688.987 | 2599.337 | 427.285 | 426.636 | 471.461 |
|  | I^-^/NO_3_^-^ | 3562.902 | 2723.95 | 2635.944 | 2717.72 | 419.476 | 463.479 | 422.591 |
|  | I^-^/ALL | 3520.972 | 2690.592 | 2666.216 | 2583.988 | 415.19 | 427.378 | 468.492 |
| Time (day) | 3 | 58.647 | 56.922 | 56.184 | 56.391 | 17.25 | 24.63 | 22.56 |
|  | 6 | 58.647 | 56.073 | 55.963 | 55.319 | 25.74 | 26.84 | 33.28 |
|  | 9 | 58.647 | 55.729 | 54.922 | 54.862 | 29.18 | 37.25 | 37.85 |
|  | 12 | 58.647 | 54.97 | 54.82 | 54.259 | 36.77 | 38.27 | 43.88 |
|  | 16 | 58.647 | 54.74 | 54.009 | 53.893 | 39.07 | 46.38 | 47.54 |
|  | 20 | 58.647 | 54.418 | 53.7 | 53.975 | 42.29 | 49.47 | 46.72 |

*Except for natural seawater adsorption assay, all other concentrations correspond to experimental values obtained after a 5-fold dilution of the tested solutions.

**Table S7.** The calculated energies of the framework for different ions.

| Ions | E_ions_ | E_interlayer_ | E(eV) | E_ions_ | E_channel_ | E(eV) |
| --- | --- | --- | --- | --- | --- | --- |
| F^−^ | -5.23327 | -1211.63 | -0.41717 | -1408.25 | -1402.71 | -0.30706 |
| Cl^−^ | -4.80205 | -1211.63 | -0.50196 | -1407.88 | -1402.71 | -0.36416 |
| Br^−^ | -4.52884 | -1211.63 | -0.52393 | -1407.62 | -1402.71 | -0.37982 |
| I^−^ | -4.25583 | -1211.63 | -0.9426 | -1407.55 | -1402.71 | -0.58283 |
| I_3_^−^ | -8.46904 | -1211.63 | -1.01855 | -1411.88 | -1402.71 | -0.70477 |

**Supplementary References**

S1. Chengkai Zhang, Zhi Wang, Wei-Dan Si, Hongxu Chu, Lan Zhou, Tong Li, Xian-Qiang Huang, Zhiyong Gao, Mohammad Azam, Chen-Ho Tung, Ping Cui, Di Sun, Dynamic and transformable Cu12 cluster-based C-H···π-stacked porous supramolecular frameworks. Nat. Commun. 2023, 14, 6413.

S2. Bin Li, Weiguang Qiu, Glenn P. A. Yap, Yves L. Dory, Jerome P. Claverie, Hydrogen-Bonded Organic Frameworks Based on Endless-Stacked Amides for Iodine Capture and Detection. Adv. Funct. Mater. 2024, 34,2311964.

S3. Yunxiao Lin, Xuanfeng Jiang, Samuel T. Kim, Sampath B. Alahakoon, Xisen Hou, Zhiyun Zhang, Christina M. Thompson, Ronald A. Smaldone, Chenfeng Ke, An Elastic Hydrogen-Bonded Cross-Linked Organic Framework for Eﬀective Iodine Capture in Water. Am. Chem. Soc. 2017, 139, 7172−7175.

S4. Mingshi Zhang, Jayanta Samanta, Benjamin A. Atterberry, Richard Staples, Aaron J. Rossini, Chenfeng Ke, A Crosslinked Ionic Organic Framework for Efficient Iodine and Iodide Remediation in Water. Angew. Chem. Int. Ed. 2022, 61, e202214189.

S5. Suman Maji, Ramalingam Natarajan, A Halogen-Bonded Organic Framework (XOF) Emissive Cocrystal for Acid Vapor and Explosive Sensing, and Iodine Capture. Small 2023, 19,2302902.

S6. Yangxue Li, Haiyang Yu, Feifan Xu, Qiaoyuan Guo, Zhigang Xie, Zhiyong Sun, Solvent controlled self-assembly of π-stacked/H-bonded supramolecular organic frameworks from a C3-symmetric monomer for iodine adsorption. CrystEngComm,2019, 21,1742–1749.

S7. Yeqing Wang, Yinying Jin, Weipeng Xian, Xiuhui Zuo, Sai Wang, Qi Sun Pore, polarity engineering in hydrogen-bonded organic frameworks for enhanced iodine capture. CrystEngComm,2019, 21, 1742.

S8. Yun Lu, Zhichao Yu, Tingting Zhang, Dingwu Pan, Jingjing Dai, Qing Li, Zhu Tao, Xin Xiao, A Cucurbit[8]uril-Based Supramolecular Framework Material for Reversible Iodine Capture in the Vapor Phase and Solution. Small 2024, 20,2308175.

S9. Qiang Li, Wenfeng Guo, Zijian Wang, Li-Li Tan, Li Shang, Tailoring Electron-Rich Fluorescent Supramolecular Organic Frameworks for Eﬃcient Capture and Visual Monitoring of Iodine. Adv. Funct. Mater. 2024,2413694.

S10. Qiang Li, Wenfeng Guo, Zijian Wang, Li-Li Tan, Li Shang, Tailoring Electron-Rich Fluorescent Supramolecular Organic Frameworks for Eﬃcient Capture and Visual Monitoring of Iodine.

S11. Yuanbo Xie, Fangyuan Zhong, Hongxu Chen, Danni Chen, Jiawen Wang, Junkuo Gao, Juming Yao, Fabrication of hydrogen-bonded metal-complex frameworks for capturing iodine. J. Solid State Chem. 2019, 277, 525–530.
